# Household Food Metabolism: Losses, Waste and Environmental Pressures of Food Consumption at the Regional Level in Spain

**DOI:** 10.3390/foods10061166

**Published:** 2021-05-22

**Authors:** Monica Di Donato, Óscar Carpintero

**Affiliations:** 1 FUHEM Ecosocial, Avda. de Portugal, 79, 2801 Madrid, Spain; 2 Research Group on Energy, Economics and System Dynamics (GEEDS), Paseo del Cauce, 59, 47011 Valladolid, Spain; carpin@eco.uva.es; 3 Department of Applied Economics, Faculty of Economics and Business, Avda. Valle Esgueva, 6, 47011 Valladolid, Spain

**Keywords:** household metabolism, food consumption, food losses and waste, water footprint, carbon footprint

## Abstract

Dealing with an increasing population is challenging the global food system not only in productive terms, but also through the associated environmental pressures. A growing diagnostic effort is being made by global and national agencies. Innovative approaches are needed to support effective policy efforts. This study aims to illustrate the potentialities of the household metabolism approach in the diagnosis of the environmental pressures derived from household food consumption, using the Spanish regions and the effects of the 2008 crisis as case studies. The direct information concerning food consumption in physical terms provided by the Spanish household budget survey is used to estimate some relevant environmental pressures (food losses and waste along the food chain, as well as water and carbon footprint) for the Spanish food system at a sub-national level. These data are directly translated into differences in environmental pressures and compared with other dietary profiles. Furthermore, the physical information of environmental pressures is related to household socio-economic status, showing the potentialities of the association with household socio-economic information. Finally, our data illustrate with some examples how the economic crisis has acted as a driver of change in food consumption, promoting a better environmental performance at the cost of poorer diets.

## 1. Introduction

Various international organisms estimate that in order to feed the projected world population of 2050, which is expected to reach almost 9600 million [1], the global food system would have to increase food availability by between 50% and 70% [2,3,4].

This a challenge of great dimensions in several different fields, the economic and the social among them, but also in the environmental, since the food system contributes in a significant way to the pressures suffered by the environment on a global level; such pressures as climate change, the loss of biodiversity, the use of fresh water, land use changes, interference in the nitrogen and phosphorous biogeochemical cycles, or chemical pollution. Thus, for instance, it has been estimated that the global food system contributes between 19% and 29% of the total greenhouse gas (GHG) emissions, which estimated to be between 9.8 and 16.9 Gt CO_2_-eq/y for 2008, in full economic crisis [5], and around 16 Gt CO_2_-eq/y in the period 2012–2016 [6]. Furthermore, the agricultural phase alone associated to the food system (food produced for human consumption or for animals destined for human consumption) implies almost 90% of the total global water footprint, which oscillated between 9.1 and 8.3 Gm^3^/y for the period 1996–2005 and the year 2011, respectively [7,8], as well as the use of almost half the ice-free land on the planet [9,10,11,12].

Recently, there has been an increase in concern over the other environmental pressure due to food consumption, which also contributes to the expansion of the other pressures [11]: food losses and waste (FLW) [13,14,15]. With good reason, among the sustainable development goals (SDG) of the United Nations for 2030 are the reduction by half of global food waste per capita in retail sales and consumption, as well as a reduction in food losses during the production and supply chains (Goal 12.3 within the Responsible Production and Consumption Goal) [16]. It has been calculated that almost 30% of the world’s food production goes to losses along the food chain, due to a lack of technical expertise on the production and supply side in the poorest countries, and a greater generation of waste in the consumption and retail sale of food in the richest countries [13,17]. In a European context, several studies have provided figures that oscillate between 173 and 290 kg per capita in FLW [13,18,19,20,21,22,23].

A great part of this environmental pressure by consumption has been laid at the door of the consumers, so it is considered that studying the household metabolism is relevant to discovering the degree of sustainability of a society [24,25,26]. In this sense, it is known that household metabolism is mainly associated, on the one hand, with food consumption and, on the other, with residential energy uses (heating, water supply, air-conditioning, refrigeration, etc.) and private transport. To be precise, some estimates show that almost 15% of energy requirements, 27% of GHG emissions or 90% of the water footprint of the households can be, directly or indirectly, attributed to their food consumption [24,25,26,27,28,29].

No single methodology is used to quantify household consumption. Although other studies use a diverse methodology [30,31,32], the majority of studies that examine household consumption are focused on entire countries or through data concerning some cities [26,33], use a top-down methodology in which the monetary quantities (the expenses of households in different categories) become physical flows (energy, food, emissions, etc.) through input-output tables and static sectorial coefficients of intensity (energy, material, emission intensities, etc.) [25,26,34]. This common approach has numerous limitations when it comes to obtaining information concerning food that can be useful for national authorities when designing policies at a sub-national scale, where the decision-making process often happens. Some examples could be the use of monetary flows to bring together the biophysical quantities, given the variability in prices and expenses that exists within each sector or category; or the limitation of scale itself that is inherent to the use of input-output tables [33]. In addition, households’ food consumption has been undergoing changes that have important environmental consequences. In some cases, these changes are more or less circumstantial, but in others they could be structural, such as those due to the consequences of the 2008 economic crisis or the tendency for many people to turn to Western diets [35,36]. To elaborate policies for the mitigation of environmental problems associated with food and make an effective diagnosis, current methodological approaches need to be complemented with biophysical information and to be produced at a more detailed scale, in which policies can actually be implemented.

Our hypothesis is that the household metabolism approach [24,25,26,34,37] could be used to complement usual monetary information and give support to policy-making. Thus, this study aims to illustrate the potentialities of the household metabolism approach in the diagnosis of the environmental pressures derived from household food consumption, using the Spanish regions and the effects of the 2008 crisis as case studies. To this purpose, biophysical information of household food consumption at a sub-national scale directly associated to socio-economic characteristics of these households has been used. Specifically, this approach has been applied to: (1) estimate the environmental pressures (i.e., losses and waste, carbon footprint, water footprint) of food consumption through the entire food chain; (2) to calculate the share of sub-national entities with varied food traditions in these environmental pressures; and, (3) to understand the responsibility of households with different socio-economic statuses in the environmental consequences of food consumption.

## 2. Materials and Methods

**Figure 1:**
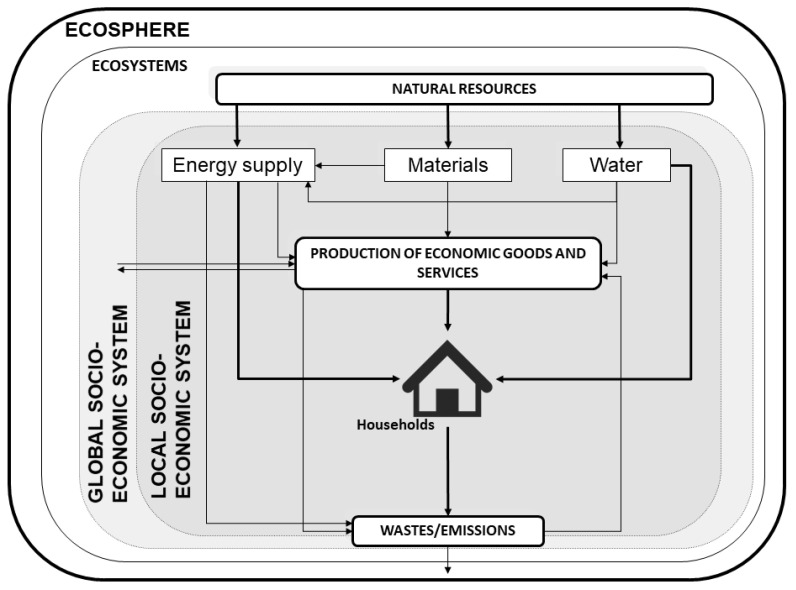
Characterization of household metabolism.

Households are defined, in line with what was stipulated by the United Nations System of National Accounts (SNA), as a socio-economic unit of consumption [38]. Here, the characterization of this socio-economic unit is made by using the household metabolism [25,26], an application of the socio-economic metabolism framework to households [37,39,40,41]. Under this approach, as shown in Figure 1, household consumption is conceived as a set of materials, water and energy (inputs, including food) flows for household maintenance and functioning; and as a result, households generate waste, emissions and effluents (outputs). At the same time, the inputs can be direct (energy, materials or water consumed directly by the household) or indirect (necessary for producing goods or services consumed directly by the household); while the same also occurs analogously with the outputs [25].

Spanish households have been grouped by region at the European NUTS-2 level (Figure S1). It should be taken into account that differences among regions are important (Table S1). Only Andalucía, C. Madrid and Cataluña account for more than 50% of total Spanish GDP. Northern regions (Aragón, Cataluña, Navarra, País Vasco, La Rioja) and the region of Madrid were over the Spanish average per capita GDP in 2012. The most populated region by far is C. Madrid, more than double the population density of the second highest (País Vasco) and over 30 times the density of the lowest one (Castilla-La Mancha) in 2012. C. Madrid, Cataluña and the islands had the highest commercial disequilibrium in 2012.

The environmental pressures calculated here concern food consumed as input to the household and waste is generated in this process. Furthermore, along the entire food chain, different resources (including water) are consumed, and emissions and losses are generated as a consequence.

Quantifying food losses and waste from households does not have a universally accepted methodology. Although there have been some attempts to standardise the calculation on a national level [42], in practice, many different approaches are still used [23]. Taking into account the lack of quality and the scarcity of direct sources of information concerning food losses and waste in Spain at both national and sub-national levels [43], for the elaboration of data for the Spanish households and regions, the direct food consumption information has been extracted from the Spanish Household Budget Survey (HBS) micro-data. This information is expressed in biophysical terms (L, kg or units) and it has been aggregated to 4 digits of the COICOP/HBS classification, statistically representative at a regional EU NUTS-2 level. This classification supposes an aggregation of the 84 original categories of food and drinks into 13 categories (Table S2). All categories were reported in kg units, by using densities from FAO/INFOODS database [44] and information on size and weight for the average egg consumption in Spain [45,46].

This information concerning actual households’ food consumption by category has been transformed into losses and waste (kg). To this purpose, coefficients have been used, in accordance with FAO evidences on losses and waste associated to the edible parts of the different HBS food categories in the food chain phases [13,14] at the European level (Table S3). Coefficients were available for the first 7 food categories, which represent the majority of fresh products, assuming that the rest have no losses or waste and adding them only at the end for the purpose of balancing the total quantity of food consumed. Thus, and according with recent calculations, the European scale for coefficients implies that the calculation method used provides only a conservative estimation of the losses and waste. In return, it is coherent with actual consumption data by category and current evidences for the losses and waste levels of different food categories at this scale.

The water footprint was calculated as the total volume of fresh water used for the consumption or the production of goods and services [29], in this case, for the consumption of food in households and the associated food production chain. To calculate the water footprint, the previously extracted data for food consumption from the HBS was used. Coefficients from literature (Table S4) were applied to estimate the water footprint, in accordance with Equation (1):

Water Footprint (L) = Coefficient (L of water/kg of food) × Food Consumption (kg).(1)

On the other hand, the carbon footprint was also calculated, understood as the GHG emissions caused, in this case, by food throughout the entire food chain, expressed in common units [47]. For the calculation of the carbon footprint, direct data previously extracted for food consumption of the HBS was used and equivalence coefficients from the literature (Table S5) in terms of units of equivalent CO_2_ (kg CO_2_-eq) (carbon footprint) per kilogramme of food consumed, according to the following equation (Equation (2)):

Carbon Footprint (kg CO_2_-eq) = Coefficient (kg CO_2_-eq/kg food) × Food Consumption (kg). (2)

Unlike the usual input-output studies, in this article, the goal is not to distinguish the origin of the environmental pressures, but to attribute a certain quantity of pressure to the consumption of each territorial entity, independently of where the corresponding phase of the food chain is carried out. The only phases of the food chain that are definitely produced in the territory to which the environmental pressures are attributed are those of retail and consumption. Thus, the direct emissions and the waste from consumption are the only ones that can be directly attributed to that territory. The rest may be the result of the passage of raw materials through the food chain and their channels of distribution.

In order to compare the footprints, three diet prototypes have been developed: the real mean diet, based on the average consumption of the different food categories of the HBS during the period 2006–2012; an adaptation of the Mediterranean diet to the Spanish context by the Spanish Society of Community Nutrition (SENC) [48], and a proposal for adapting this diet to an ovo-lacto-vegetarian (OLV) diet [49]. These last two have been adapted to the HBS categories for the sake of comparison. Then the abovementioned coefficients were used to calculate the water and carbon footprints corresponding to those diet prototypes. More details concerning the elaboration of the three diets (rations, intake used, etc.) can be consulted in the Supplementary Materials (Tables S6 and S7).

Finally, to understand the potential of socio-economic information, the environmental pressures by total per capita expenditure levels were calculated. Income and expenditure data provide different information. However, often an underestimation of income data in HBS was detected. Consequently, data concerning income in these types of surveys are less reliable, and do not have the same quality as the data concerning expenditure. Thus, it is a usual and widely accepted practice among researchers to use total expenditure as a proxy for income [50,51,52]. According to literature, this is the case for the Spanish HBS [53,54,55]. The economic rationale of this assumption is that, for surveys like HBS, consumption better represents the household economic status than income, which is more robustly estimated in other sources, such as, e.g., the Survey of Income and Living Conditions (SILC).

## 3. Results

### 3.1. Losses and Waste in the Food Chain 

On the basis of the data concerning the consumption of food by categories extracted from the HBS (Table 1), Table 2 shows the estimate of the per capita quantity of losses and waste generated in each stage of the food chain over the period 2006–2012 in the NUTS-2 regions of Spain.

For the whole of Spain, it has been estimated that between 44% and 49% of the total amount of municipal urban waste is organic, mainly from food consumption in households [56]. This large volume of waste is reflected in the weight that household food consumption has within the losses and waste generated throughout the entire food chain (Table 2; Figures S2–S4). In Spain, almost 35% of total food losses and waste corresponds to the phase of household consumption in the period 2006–2012, with values that oscillate between 2.7 and 2.9 Mt of total waste, which corresponds to levels of 61.7 and 66.1 kg per capita at the start and end of the period. Next comes the estimated losses for the agricultural production phase, which represents a little over 33% of the total (between 57.7 and 61.7 kg per capita in the studied period), with lesser weights of 15% for the rest of the phases in the food chain (post-harvest handling, processing and packing and distribution). Other sources at national level offer figures for the period of the crisis studied, that oscillate between 1.5 and 2.9 Mt of food waste from households in Spain as a whole [57,58].

Nevertheless, there are differences between the NUTS-2 regions (Table 2). These depend on the weight that each category has in the total food consumption. Higher levels of waste are to be found associated with the households of the northern regions, in particular those of the north-west, especially the households of Galicia with levels of waste associated to consumption of over 75 kg/inhabitant in 2008, the households of Asturias that reached 72 kg/inhabitant in 2010 or those of Castilla y León that almost reached 72 kg/inhabitant in 2008. On the other hand, it is the households of the Balearic Islands and the Islas Canarias (55.6 and 53.4 kg/inhabitant, respectively), as well as the Mediterranean regions, that present the lowest levels of waste associated to consumption.

These patterns, logically, reappear in the phases of the food chain associated with the agro-food industry, where the households of Galicia and Castilla y León once more appear at the top of the regional classification, followed by the households of Aragón and Navarra; while the households of the Islands and Extremadura finish the list, with the lowest waste associated with consumption within these phases of the food chain. As for agricultural production, only the households of Galicia pass 70 kg/inhabitant of losses associated with consumption. The decreases in food consumption that occurred during the crisis resulted in Extremadura being the region where households had the lowest levels of losses associated with agricultural production (52 kg/inhabitant), followed by Islas Canarias (52.3 kg/inhabitant).

Concerning the temporal evolution, in the period under study, there was a peak in losses and waste associated with the food chain between 2008 and 2010, coinciding with the peak in consumption. From then on, due to the effects of the crisis on household consumption, there was a slight reduction in losses and waste in the food chain. Thus, in 2012, the levels were at the same levels as in 2006 or 2008, as far as waste and losses were concerned in most phases of the food chain. On average, in Spanish households as a whole, there was a reduction of almost 2% in the losses and waste associated with the food chain in the period 2006–2012, although waste once more took on an ascendant path for 2009 and 2012, with a slight upturn of 0.32%.

This temporal evolution of Spain’s average household is a fairly generalised pattern among the households of the different regions (NUTS-2). The reduction in the weight of losses and waste with respect to the food flow over the entire period is particularly relevant in the households of Andalucía, Islas Baleares and Castilla La Mancha, reaching a 5% improvement. On the other hand, it can be found that the households of Aragón, Galicia, Murcia, Navarra and the Basque Country, where there are increases in the weight of losses and waste with respect to the flow of food that in no case is over 3.2%. The effect of the crisis was also noted when the households of over half the regions increased the weight of waste and losses within the food flow between 2009 and 2012. This effect is stronger in the households of Aragón, C. Valenciana and the R. Murcia, where there are increases of 2.44%, 3.53% and 3.0%, respectively. The households of Asturias, the Islands, Cantabria, Castilla La Mancha, Galicia and País Vasco continue to show a downward trend.

### 3.2. Carbon Footprint

**Figure 2:**
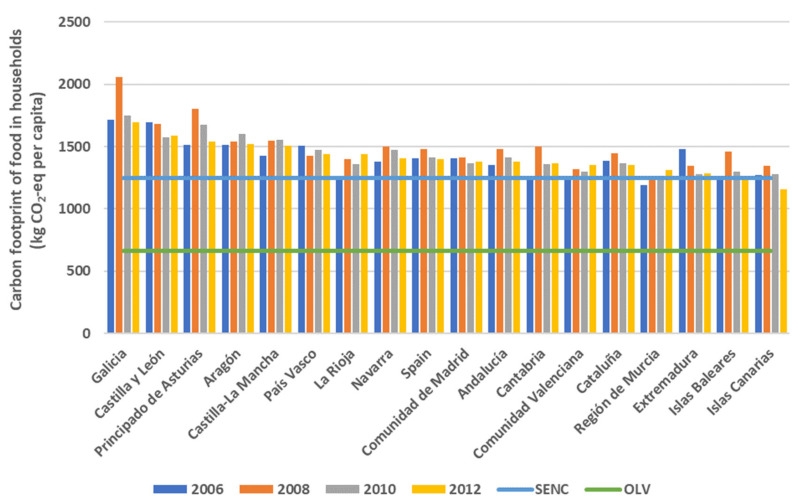
Estimation of carbon footprint of Spanish households by NUTS-2 region (including Spanish average) and carbon footprint levels for Spanish Society of Community Nutrition (SENC) and ovo-lacto-vegetarian (OLV) healthy diets (kg CO_2_-eq per capita) in the period 2006–2012.

As already mentioned, the treatment of emissions associated with food has been done through the carbon footprint indicator. In accordance with the methodology used here (see Table S4 and Equation (2)), on average, the food consumption of a Spanish household between 2006 and 2012 involved emissions of 1.4 t CO_2_-eq/inhabitant per year (Figure 2).

As shown, although there was an increase in total emissions associated with the food consumption of Spanish households of 5%, over the entire period, going from 61.4 Mt to 64.5 Mt CO_2_-eq, the average intensity of consumption per capita grew by 5.6% until 2008 (from 1.403 to 1.481 kg CO_2_-eq/inhabitant). This increase was later compensated for in part by a fall due to the general decrease in household food consumption that occurred during the economic crisis, suffering, in net terms during the entire period, only a slight increase from 1.403 to 1.427 kg CO_2_-eq/inhabitant (0.5%). These figures are close to the calculations carried out in other works, which consider the whole food chain for Spain in [59].

This general tendency for Spain as a whole is more or less similar to that generated in the households of the majority of the regions (Figure 2). Thus, the food carbon footprint for the households of central-northern Spain (with a tendency of being more rural) shows levels that are above the average for Spain, reaching values of 1.8 t CO_2_-eq per capita in Galicia, and more than 1.6 t CO_2_-eq per capita in Asturias or Castilla y León. Vice versa, it is the regions of the south and the Mediterranean, as well as the Islands, which have lower levels of the carbon footprints, with values close to 1.2–1.3 t CO_2_-eq per capita.

One possible explanation would be that the central-northern households, in general, have diets with a greater presence of proteins of animal origin that come from dairy produce, meat and fish and which, in many cases, make up part of the traditional production of these regions. Thus, the increase in the consumption of meat, milk, dairy produce and eggs in this period (around 14%), despite the fall of 3% in consumption per capita of fish, meant that, taking all the categories as a whole, the total was over 73% of the estimated carbon footprint in 2012 (1.396 kg CO_2_-eq/inhabitant) for Spain’s households as a whole.

**Figure 3:**
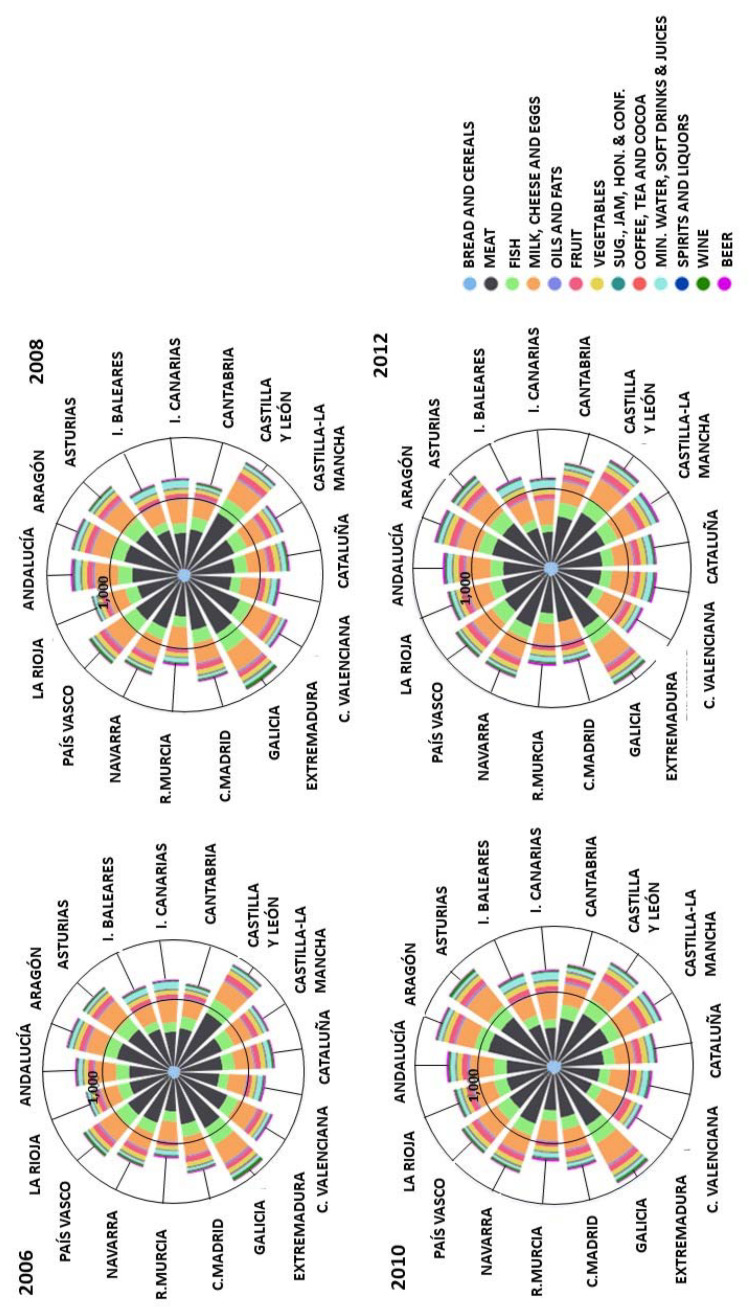
Share of carbon footprint by food categories (COICOP/HBS 4 digits) for each NUTS-2 region (kg CO_2_-eq per capita).

If the weight of each category in the household food carbon footprint region by region is examined (Figure 3), it is in households of such regions as Galicia or Asturias, and also those of Aragón and Castilla y León, due to a greater consumption associated with products of animal origin, where the household food carbon footprint is greater and the relapse is especially high. On the other hand, the households of the Mediterranean regions and the Islands show lower per capita emission levels associated with food consumption, since they are closer to the Mediterranean diet, with a greater presence of foods and fat of a vegetable origin, which are less intensive in emissions; although, in the profile of their food emissions, the categories of meat, fish, dairy produce and eggs still predominate.

The increases in consumption of alcoholic and soft drinks, as well as sugar and sweets, have also generated an increase of their weight in the carbon footprint, oscillating between 11–12%, 20% and 10%, respectively, for Spanish households as a whole. This increase for soft drinks and beer occurred mainly in households of the Mediterranean coastal regions and the Islands (Andalucía, Cataluña, Valencia, Murcia, Islas Baleares and Islas Canarias). This may be associated with the Mediterranean climate, which is hotter; the hardness of water and its contamination, in the cases of mineral water, soft drinks and fruit juices; as well as to the regional economy’s dependence on tourism in the case of beer. As for wines, it is the northern regions (Galicia, Asturias and Navarra) where the carbon footprints derived from their consumption are greatest. Finally, considering sugar and sweets, it is also the northern households that show the greatest increase in the associated carbon footprint due to the increase in consumption, especially in the households of Asturias and Navarra.

**Figure 4:**
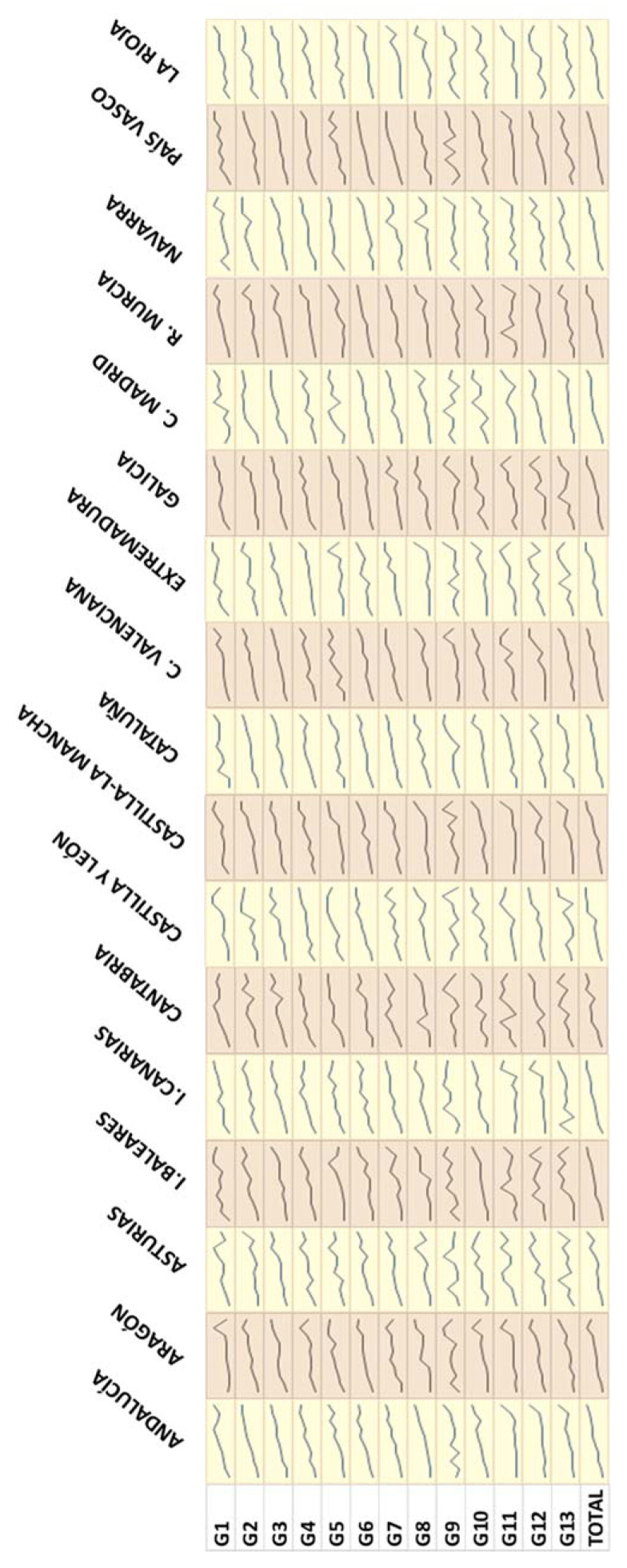
Trends in average values of carbon footprint by decile of total household expenditure for the different food categories and NUTS-2 regions in 2012 (kg CO_2_-eq/inhabitant). G1 = bread and cereals; G2 = meat; G3 = fish; G4 = milk, cheese and eggs; G5 = oils and fats; G6 = fruit; G7 = vegetables; G8 = sugar, jam, honey, chocolate and confectioneries; G9 = coffee, tea and cocoa; G10 = mineral water, soft drinks and juices; G11 = spirits and liquors; G12 = wine; G13 = beer.

However, carbon footprints are also distributed unequally according to the economic situation of the households. For all the years in the series (Figure 4 shows 2012), it can be seen that, for all the regions, the households of the deciles with the highest total expenditure per capita are those which also have the highest carbon footprint per capita in all the food categories. There are some exceptions, where the last decile in expenditure has, on average, lower carbon footprints than the first decile, but all the rest show the same pattern; thus, it can be said that it is a generalised occurrence. In 2012, the exception occurs in the case of coffee, tea and infusions (G9) in Castilla y León, where the households in the last total expenditure decile have a carbon footprint per capita which is 63% lower than that of the first decile. However, confirming the general tendency, the ninth decile in total expenditure per capita has a carbon footprint per capita 54% higher than that of the first decile.

Therefore, the values of the per capita footprint increase in line with the total expenditure of the households, oscillating according to region and category. Considering the total food for each region, the smallest difference in the per capita carbon footprints can be seen in the households of C. Madrid, where there is an increase of nearly 51% between those of the highest and lowest spending deciles; while the greatest difference between both can be seen in the households of La Rioja, where this value increases to a little over 70%. This pattern of behaviour of the per capita carbon footprint is connected to the highest levels of per capita consumption in the households in the highest deciles of total expenditure. That is to say, as is logical, the higher the consumption, the higher the carbon footprint, especially if the food consumption is linked to certain food categories that are more intensive in emissions, such as all those rich in proteins of an animal origin.

As for the adoption of other dietary patterns by Spain’s households, these would present room for improvement in terms of the carbon footprint (Figure 2). This would depend, apart from on the consumption itself, on the degree of exterior dependence (transport), the type of cultivation and other conditioning factors concerning the means of production and consumption. Thus, a healthy omnivorous dietary pattern such as that of the SENC would imply an average in emissions associated with food consumption close to 1.2 t CO_2_-eq/inhabitant. Adopting this type of dietary pattern would reduce the carbon footprint of food consumption in Spain’s households by something over 12% with respect to the average level during the studied period. As for the OLV healthy dietary pattern, this would imply an average carbon footprint of 661 kg CO_2_-eq/inhabitant; thus, were it to be adopted, the possibilities for reduction would be almost 54% with respect to the real average dietary level of Spain’s households in the studied period. As is logical, on the basis of what has been stated here, the margins for improvement would be greater in the households of regions where the diets involve the consumption of more components of an animal origin, as this could be reduced to a greater extent.

### 3.3. Water Footprint

Apart from the associated emissions, food also involves the indirect use of water derived from the production process, the distribution throughout the food chain and the consumption by households. In order to capture this indirect consumption of water, the water footprint indicator was used [7,60]. Thus, in accordance with the methodology used here (see the Table S2 and Equation (1)), the average consumption of food by Spain’s households in the period 2006–2012 involved an average water footprint of 1424 m^3^ of water/inhabitant.

**Figure 5:**
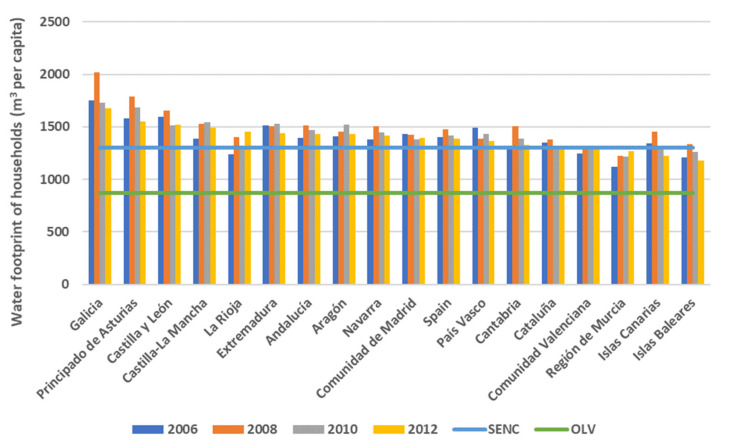
Estimation of water footprint of Spanish households by NUTS-2 region (including Spanish average) and water footprint levels for SENC and OLV healthy diets (m^3^ per capita) in the period 2006–2012.

As for its evolution, the total average water footprint from food consumption in Spain’s households has grown by 4.8% between 2006 and 2012 (from 61.3 to 64.3 km^3^), associated with the tendencies in food consumption and the gradual change in diet that has been occurring [61,62]. Thus, every Spanish person has seen how the level of their per capita water footprint associated with food consumption, following an increase of over 5% between 2006 and 2008 (from 1401 to 1473 m^3^ of water/inhabitant), was compensated until it was reduced by 0.7% in the entire period (1391 m^3^ of water/inhabitant in 2012) (Figure 5), due to the decrease in the total food consumption that took place during the years of the economic crisis.

**Figure 6:**
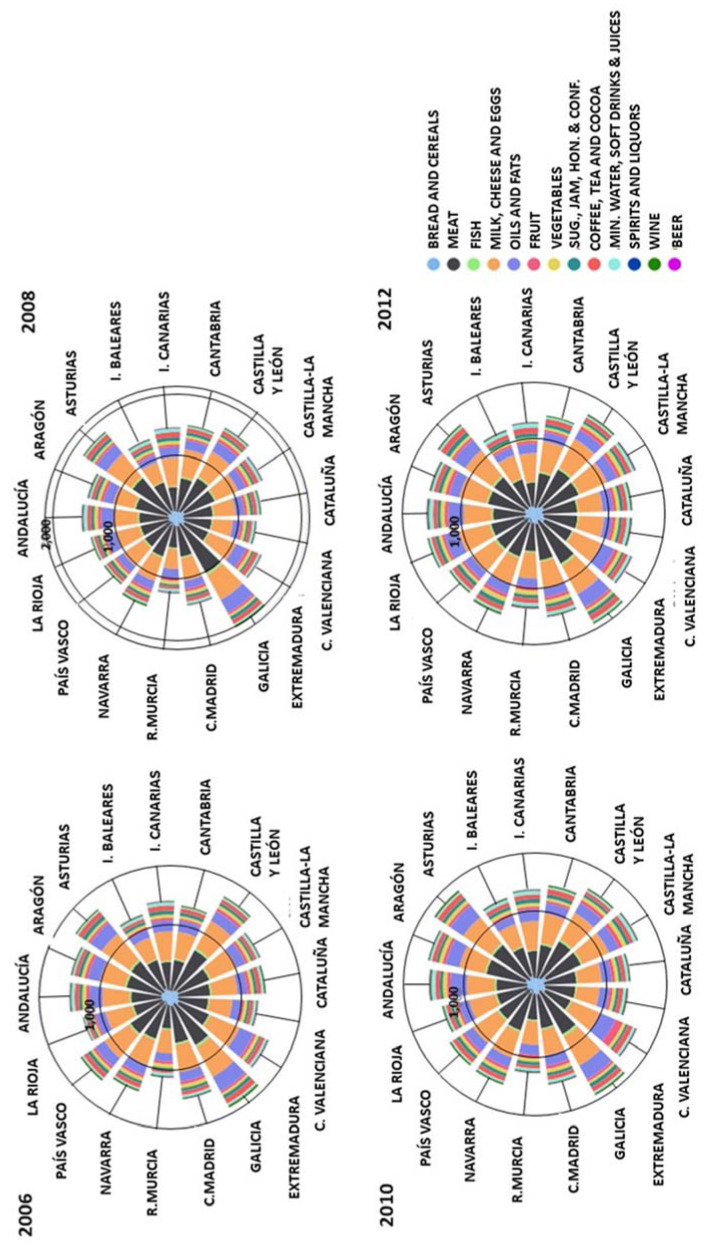
Share of water footprint by food categories (COICOP/HBS 4 digits) for each NUTS-2 region (m^3^ per capita).

The share of the food per capita water footprint for households according to the regions (NUTS-2) is also unequal (Figure 6). It shows that the households whose water footprint is greater than the Spanish average are in the northern regions (Galicia and Asturias at the top), the continental regions of Spain and some southern regions (Extremadura and Andalucía), which partially coincides with the predominance of the consumption of food linked to products of an animal origin (meat, dairy and eggs, as well as oils and fats). These households have annual water footprints from food consumption with values between 1600 and 1800 m^3^/inhabitant. The households of the Islands and all the Mediterranean regions, except Andalucía, are in the lower part of the classification concerning the water footprint, given the type of food consumption they have, which is based more on products of a vegetable origin, with a lower associated water footprint. The results in this group of regions show annual water footprint levels that originate in food consumption range from 1200 to over 1300 m^3^/inhabitant.

If the weight of each category in the per capita water footprint by NUTS-2 region (Figure 6) is examined, the categories of meat, dairy and eggs, and oils and fats still predominate in all the regions throughout the entire period studied. They estimate an average of 71% of the per capita water footprint of Spain’s households as a whole. Worth noting are the water footprints associated with these categories in the households of some northern regions, such as Galicia, Asturias or Cantabria, where the percentage passes 74% of the total water footprint from food for 2012. On the other hand, households of the Mediterranean regions and the Islands were those where these three categories did not reach 70% of the total water footprint from food.

**Figure 7:**
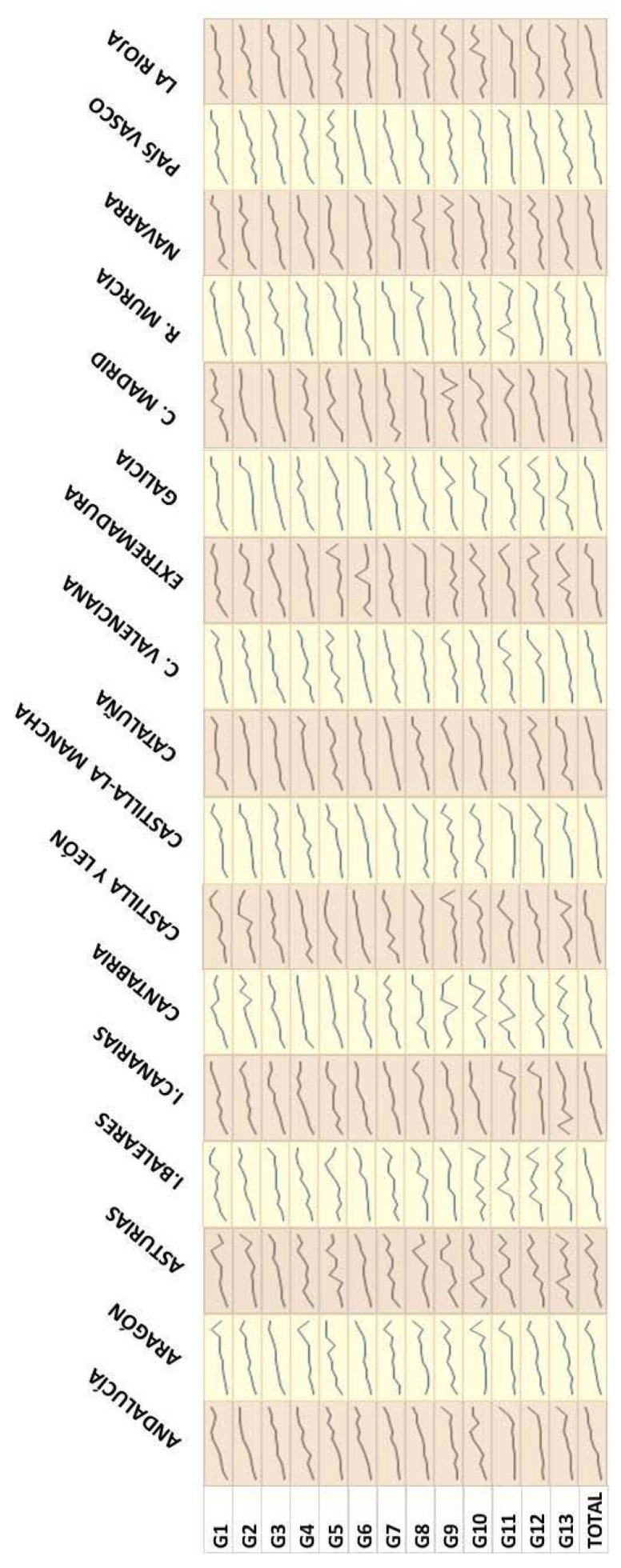
Trends in average values of water footprint by decile of total household expenditure for the different food categories and NUTS-2 regions in 2012 (m^3^/inhabitant). G1 = bread and cereals; G2 = meat; G3 = fish; G4 = milk, cheese and eggs; G5 = oils and fats; G6 = fruit; G7 = vegetables; G8 = sugar, jam, honey, chocolate and confectioneries; G9 = coffee, tea and cocoa; G10 = mineral water, soft drinks and juices; G11 = spirits and liquors; G12 = wine; G13 = beer.

On the other hand, the per capita water footprint also presents differences between more or less well-off households in the different regions. Thus, as in the case of the carbon footprint, the conclusion can also be easily reached here that, although it oscillates for many regions, the richest households, those belonging to the deciles with the highest expenditure, present the highest per capita water footprints for every year, food category and region. One example of this general tendency can be observed for the year 2012 of the period under study (Figure 7). The range of increase in the water footprint per capita among the most extreme deciles for total expenditure (the households belonging to the first decile as opposed to those of the last decile) go from 85 to 219%, seeing food as a whole within households at a regional level. However, this range is even larger for some specific categories, such as spirits and liquors (G11), where differences are over 2000% in the regional households of Asturias, Madrid, Navarra or the Basque Country. Once more, there are a few exceptions (the case of the region of Cantabria in 2012, for instance), where the level of water footprint per capita is lower in the decile with the largest per capita total expenditure as opposed to that of the lowest (water footprint 27% lower in this case) in one category (that of stimulants (G9)).

Nevertheless, the tendency for growth in the per capita water footprint is maintained until the deciles with expenditures immediately below that of the highest; this allows us to state, with no fear of being mistaken, that this pattern also occurs in those food categories and regions.

As in the case of the carbon footprint, the water footprint of Spain’s households could also be reduced considerably by adopting more healthy dietary patterns (Figure 5). In accordance with the methodology adopted here, the food categories of the HBS and the coefficients used (see the Table S5), the average water footprint per capita for a healthy omnivorous diet such as that of the SENC would be approximately 1303 m^3^/inhabitant. This would suggest a margin of improvement with respect to the average annual intake of Spanish households in the period 2006–2012 of 8.5%. Nevertheless, the average per capita water footprint of an ovo-lacto-vegetarian diet in a Spanish context would be 867 m^3^/inhabitant (see Table S6). This would allow a much greater margin of improvement with respect to the average annual intake of Spanish households, which in turn would allow a reduction in the water footprint of a little over 39%. As is logical, households of regions where the consumption of meat, eggs and dairy, and oils and fats (which represents an average of 71% of the water footprint) is higher would also have wider margins for improvement through the reduction of this type of food consumption and the adoption of another type of diet.

## 4. Discussion

Currently, proposals to reduce FLW and associated environmental pressures are mainly focused on [63,64,65]: (1) implementing prevention initiatives in production and consumption; (2) measuring and monitoring; (3) assessing benefits, costs and trade-offs; (4) designing policies under limited information; (5) understanding the systemic effects along the food chain and across countries; and (6) considering the effects of socio-economic changes in demand. In this sense, the main initiatives developed to reduce food losses and waste at the Spanish national scale have been focused on [66]: (1) monitoring losses and waste from households and surveys to some small and medium-size enterprises (SMEs) from industry; (2) spreading of good practices and collaboration agreements with some food SMEs; (3) redistribution of food surplus to charities and NGOs; and (4) food technological agreements.

The metabolic approach applied here, together with direct information from sources like HBS, can be used to support some of these specific policies focused on reducing food losses and waste, and environmental pressures associated.

Apart from direct biophysical data of consumption, the use of HBS to deal with the consumption stage of food metabolism allows for the analysis of socio-economic factors in relation to food consumption and associated environmental pressures. In this case study, as the economic literature points out, the context of rising prices, decreasing income, high unemployment rates and labour instability, such as occurred in the economic crisis of 2008, had the effect of slowing down household consumption [67,68]. It also, therefore, had an effect on the tendencies prior to the rise in consumption of many food categories that could still be appreciated in the data corresponding to the period 2006–2008. This contraction in consumption led to a reduction in losses and waste generated throughout the food chain, as well as a decrease in the food footprint in the different dimensions dealt with here. The contraction was on occasion strong and lasted for the entire period under study. On other occasions, a slight comeback started, but one which was not sufficient to compensate for the previous contraction in most cases. However, the said reduction did not occur as a result of an improvement in the efficiency of the food chain or through a change to a more sustainable dietary pattern, as already pointed out by other authors using diverse sources [69]. It was rather the result of the reduction in total expenditure on food that this unstable economic period and all the negative socio-economic consequences produced in Spanish households, that lowered the level of food safety and worsened their diets [70,71]. There is a clear association between the economic status of households and footprint levels for most food categories and Spanish NUTS-2 regions. In all of them, the richest households continue to have a predominance in food consumption levels and associated environmental pressures. The difference between levels were not only reduced but also increased during the period. This probably happened because the crisis affected households differently according to their economic status. Thus, the richest households could only reduce their consumption of other goods and services, but the poorest households were also forced to adjust their consumption of food, and therefore the associated pressures.

HBS could be a powerful tool for the direct analysis of these relationships and a check of causal relationships between socio-economic factors and food consumption patterns. It could lead to more effective and focused environmental policies on certain socio-economic groups according to their socio-economic status or the characteristics of households or individuals [72,73,74,75].

Unfortunately, despite all these reductions in the levels of environmental pressure associated with food consumption in Spain due to the economic crisis, it would seem that the levels of environmental pressure remain in the mid and mid-high part of the classification with respect to other European countries.

Thus, for losses and waste from the food chain, the levels estimated here for the whole of Spain would be close to those estimated by the Barilla Center for Food and Nutrition (BCFN) [22]. This represents levels above the estimations carried out for southern European countries such as Italy, Portugal or Greece (149, 132 and 44 kg/inhabitant, respectively), but also below the estimates carried out for such central northern countries such as The Netherlands, the UK or Finland (579, 238 and 193 kg/inhabitant, respectively).

As for the food carbon footprint, comparing the average of the Spanish households’ emissions in the period 2006–2012 with the data of other similar studies for this period, it can be seen that the levels of emissions associated with food consumption are below those of households of other countries of northern Europe, such as Germany and Ireland, but above those of such countries as China or the USA [76,77,78,79]. Taking as a reference that a typical Mediterranean holm oak woodland (*Quercus ilex*) can absorb between 3–12 t CO_2_/ha/y [80], in order to compensate for this level of emissions, between 5.4 and 21.5 Mha of Mediterranean woodland would be needed (between 11 and 43% of the entire surface of the country).

In the case of the water footprint, the average level found in Spain is between the values associated with food consumption of the central and eastern European Union countries [81], but far below the values of the southern EU countries within this same work, and even below the levels attributed to such countries as the USA [82]. Nevertheless, the levels are still very high since, for the period 2006–2012, they estimate something over 27 times the average consumption of water for domestic purposes (hot water, toilet, cooking, cleaning, etc.) registered by the same HBS for that period in those same households (51.8 m^3^ of water/inhabitant).

Comparison of actual food consumption and environmental pressures associated with different hypothetical dietary profiles confirms that simply changing some food habits, especially the consumption of animal products, can produce improvements in the reduction of environmental pressures along the entire food chain, with synergic health effects [83,84,85].

However, our results also illustrate the fact that apart from consumers, a significant amount of losses and waste are associated with other stages within the food chain. Additionally, recent trends in the reduction of FLW involve more than mere consumption, e.g., the reuse of food waste in agricultural production [86]. It is necessary to appropriately monitor and quantify them. The metabolism approach can be used to trace the origin, distribution, consumption and their respective losses and waste, but it requires a life cycle approach to statistics linked to the food chain [32].

The lack of quality information concerning the waste statistics throughout the entire food chain hinders the possibility of extracting direct information with which to follow the pressures, losses and waste at each of the nodes in the food chain, including countrywide in the case of Spain [43]. To go beyond these limitations, a consumption-based perspective has been chosen, that is, starting from the consumption of the different food categories (directly obtained information) and going backwards towards the estimates of related losses and waste along the entire food chain [25]. The inconvenience is that the geographical origin of the food is not known, and thus the possibility of understanding the territorial distribution of the pressures, losses and the waste throughout the entire food chain. Given the extent of the food chains and the multitude of intermediate stages that exist for many products between the producer and the consumer, it is a huge challenge to trace the origin of products that correspond to the different food categories along the chain, extracting the respective associated pressures in each node. All this is further complicated when the information gathered is direct (biophysical).

Another relevant aspect of the diet that is not considered here concerns the food intake outside of the home. The Spanish HBS counts this consumption simply as another monetary expense of households, without considering the physical quantities that correspond to the food and drink consumed by households. Other statistics take it into account in biophysical terms, but it is impossible to cross reference them with the socioeconomic data of the household to which the individuals who carry out the said consumption belong. This means that there are fewer possibilities of understanding the phenomenon of food consumption outside the home or being able to compare it in a homogeneous way with the food consumed directly by the household.

However, this reduction in the environmental pressure of the food chain was not homogeneous throughout Spain. Rather, for most fresh products, there was a gradient that went from predominantly rural and central northern regions (these generally consumed more products of animal origin and therefore had greater associated pressure) to southern and Mediterranean regions or the Islands, with a different type of consumption which notably reduces the levels of the food associated footprints, as well as the per capita losses and waste generated. However, for most beverages, especially those associated with leisure time, the gradient was inverted, and it was the central southern regions, the coastal areas and the Islands which, in general, presented greater environmental pressure associated with this type of consumption. This presupposes slightly different dietary patterns with different consequences in terms of environmental pressure. Thus, for conscious environmental food policies, the central northern regions of the country enjoy advantages insofar as there are short food chains in these areas, due to productive traditions associated with the Atlantic diet [87,88]; but they also have the disadvantage of greater associated environmental pressures due to a higher consumption of animal products. Meanwhile, the central southern regions and the Islands have a somewhat better consumption from an environmental point of view, but with a greater consumption of some less recommendable categories from the point of view of health. It would also be necessary to check up to what point these gradients have an economic origin, i.e., whether less food is consumed due to environmental reasons, food traditions or productive culture, or just economic ones.

A diagnosis of the differences in food consumption and environmental pressures in households at a sub-national level may turn out to be highly relevant in the design of effective public policies. It may serve as a link between the directives proposed on a global, regional or national level and the diagnostic work developed in cities concerning the urban metabolism [63,75,89]. Thus, it is possible to spell out the former within the territory and to provide the latter with a context within the articulation of the competences that each state has in the management of the different steps in the food chain, which normally fall outside the domain of any particular city.

## 5. Conclusions

In the efforts to make a reliable and relevant diagnosis of the food contribution to the environmental pressures, the household metabolism can be complementary to the usual monetary approaches.

In this article, the case study of Spain and its regions shows how the analysis of household metabolism together with HBS can provide valuable information in terms of the environmental pressures of food consumption. Thus, the increase of the FLW and carbon and water footprints experienced in the previous economic accumulation cycle was interrupted by the 2008 economic crisis outbreak, but the socio-economic drivers of this phenomenon did not disappear. The inequality growth experienced during the crisis has potentially acted as a powerful driver since the relationship between the economic status and the environmental pressures was clear: the richest households were responsible for higher environmental pressure levels. Furthermore, the use of a sub-national scale (NUTS-2 regions), where the decision levels regarding the food chain are often found, has completed the vision, at times limited, of the urban metabolism and allows us to potentially support a more focused range of food policies on a national level. Finally, our data also support the conclusion that it is possible to decrease the environmental pressure of food consumption just by making some changes to diets, specifically, reducing the consumption of animal products.

However, this approach implies different limitations. One is associated with the lack of quality data on FLW, partially based on consumption at the global and national levels, but with numerous gaps in the rest of the food chain, and at a subnational level. Furthermore, there is a need for a comprehensive framework to include agrarian, industrial and distributional stages in a coherent way to support a metabolic view of this process.

Further research must cover these and other gaps, and also investigate the social and economic drivers of food consumption patterns, in order to be capable of supporting effective policies to deal with environmental problems associated with the food system.

## Figures and Tables

**Table 1:** Food consumption (kg per capita) by NUTS-2 regions in Spain.

NUTS-2 Region	2006	2008	2010	2012
Andalucía	637.8	709.5	693.5	678.5
Aragón	636.1	677.3	659.8	661.4
Asturias	648.6	685.7	694.1	652.7
Islas Baleares	647.0	713.3	719.7	682.9
Islas Canarias	683.8	742.6	705.9	673.1
Cantabria	554.3	644.3	633.2	600.9
Castilla y León	668.3	703.2	663.1	681.9
Castilla-La Mancha	632.0	699.6	714.5	706.1
Cataluña	647.5	677.6	666.4	649.3
C. Valenciana	619.6	655.1	660.8	671.5
Extremadura	580.3	624.8	642.5	618.3
Galicia	700.3	762.7	716.9	719.4
C. Madrid	578.9	606.5	598.1	598.4
R. Murcia	580.2	650.2	633.0	661.7
Navarra	574.6	641.6	594.2	631.0
País Vasco	605.7	585.8	612.6	592.8
La Rioja	543.3	623.8	619.6	652.0
Spain	630.2	699.5	698.4	693.7

Source: HBS. Baseline 2006 (INE).

**Table 2:** Losses and wastes associated with the different stages of the food chain in Spanish households by NUTS-2 regions (kg per capita).

	Agricultural Production				Post-Harvest Handling and Storage				Processing and Packaging				Distribution				Consumption			
	2006	2008	2010	2012	2006	2008	2010	2012	2006	2008	2010	2012	2006	2008	2010	2012	2006	2008	2010	2012
**Andalucía**	55.63	58.98	59.10	56.31	14.23	14.97	15.16	14.37	19.46	20.85	20.70	19.99	20.77	21.88	21.57	20.56	62.02	64.52	64.61	61.59
**Aragón**	61.15	63.13	63.79	66.32	14.87	15.71	16.00	16.31	20.35	21.33	22.06	21.60	22.53	23.17	23.56	24.22	62.31	65.62	67.34	67.18
**Asturias**	63.65	66.79	70.88	62.10	15.53	16.13	17.15	15.16	21.21	22.56	23.29	20.43	23.34	24.74	25.67	23.11	67.40	70.40	72.19	66.81
**I. Baleares**	52.86	56.96	59.70	53.79	13.77	14.66	15.26	13.64	18.27	19.70	19.86	17.85	19.02	20.64	21.52	19.15	56.79	60.54	62.01	55.55
**I. Canarias**	53.31	57.98	55.90	52.38	13.20	14.53	13.88	13.21	18.00	19.79	18.43	17.40	18.51	19.93	19.25	18.08	54.56	59.90	55.77	53.58
**Cantabria**	52.33	61.39	57.76	57.09	13.03	15.24	14.34	14.08	17.48	20.79	19.54	19.08	19.16	22.48	21.15	20.96	57.02	66.12	62.67	61.12
**Castilla** **y León**	67.22	68.05	67.77	68.33	16.21	16.46	16.25	16.55	22.12	22.34	21.59	21.59	25.12	25.33	25.24	25.42	70.29	71.71	69.34	71.01
**Castilla-La Mancha**	58.02	60.79	64.69	61.03	14.66	15.73	16.69	15.54	19.97	21.33	22.13	21.31	21.65	22.72	24.11	22.69	64.02	68.37	71.83	67.47
**Cataluña**	59.63	59.50	60.17	57.58	14.96	15.02	15.08	14.43	19.84	20.14	19.65	19.23	21.81	21.76	21.83	21.01	62.18	62.45	61.56	59.41
**C. Valenciana**	50.69	52.74	54.92	54.90	12.96	13.62	14.11	14.04	17.49	18.58	18.93	18.84	18.46	19.23	19.89	20.09	54.83	57.68	58.31	59.07
**Extremadura**	49.18	53.35	60.56	51.96	12.52	13.72	15.08	13.50	17.80	18.85	19.05	18.41	18.45	19.69	22.15	19.19	57.92	62.37	65.36	60.78
**Galicia**	67.42	74.09	72.12	72.46	16.46	17.98	17.35	17.65	23.72	26.73	24.52	25.06	24.84	27.75	26.71	26.54	71.19	77.65	73.59	74.04
**C. Madrid**	57.61	58.20	58.86	58.21	14.31	14.66	14.75	14.68	19.19	19.40	19.17	19.24	21.41	21.63	21.80	21.67	61.47	62.99	62.30	62.56
**R. Murcia**	47.66	54.01	52.30	54.94	12.45	14.37	13.96	14.34	17.00	19.39	19.09	19.76	17.44	19.50	18.95	19.84	53.12	60.82	59.26	59.98
**Navarra**	59.54	66.35	60.86	66.89	14.99	16.63	15.31	16.72	19.98	21.79	20.44	21.77	21.66	24.12	22.48	24.13	62.91	69.67	65.00	68.59
**País Vasco**	63.54	61.68	65.30	62.78	15.29	15.02	15.96	15.39	20.31	19.93	21.07	20.15	23.35	22.64	24.01	23.35	65.47	64.20	67.68	65.72
**La Rioja**	50.92	57.58	56.17	58.27	12.78	14.32	14.22	15.05	17.49	19.74	18.90	20.73	18.78	21.05	20.84	21.34	56.69	62.17	62.37	66.51
**Spain**	57.66	62.12	63.82	62.24	14.43	15.63	16.03	15.64	19.59	21.35	21.41	21.07	21.23	22.85	23.33	22.80	61.73	66.70	67.31	66.12

## Data Availability

This work has been elaborated with public and anonymous micro-data from the Spanish HBS, which can be found on the Spanish National Institute of Statistics (INE) website: https://cutt.ly/sb8Ulfe (accessed on 20 May 2021).

## Supplementary Materials

The following supplementary materials are available online at https://www.mdpi.com/article/10.3390/foods10061166/s1, Figure S1. Location of EU-NUTS-2 regions in Spain; Figure S2. Distribution of losses and waste by food chain phase and NUTS-2 region in Spain (kg per capita) for 2006; Figure S3. Distribution of losses and waste by food chain phase and NUTS-2 region in Spain (kg per capita) for 2009; Figure S4. Distribution of losses and waste by food chain phase and NUTS-2 region in Spain (kg per capita) for 2012; Table S1. Some socio-economic characteristics of NUTS-2 regions in Spain; Table S2. Aggregation of COICOP/HBS categories used in this work; Table S3. Coefficients used to calculate losses and waste associated to consumption of main food categories; Table S4. Water footprint of 5-digit COICOP/HBS food categories used (L/kg); Table S5. Carbon footprint of 5-digit COICOP/HBS food categories used (kg CO2-eq/kg); Table S6. Parameters of frequency and weight of daily recommended portions, and the resulting annual intake calculated for an omnivorous healthy diet; Table S7. Parameters of frequency and weight of daily recommended portions, and the resulting annual intake calculated for an ovo-lacto-vegetarian healthy diet.
