# Supplementary material for: Household Food Metabolism: Losses, Waste and Environmental Pressures of Food Consumption at the Regional Level in Spain
Source: Foods. 2021 May 22;10(6):1166. doi: 10.3390/foods10061166 (PMC8224788; doi:10.3390/foods10061166)
Supplement: Supplementary file 1 [file foods-10-01166-s001.zip › foods-1233723Suppltmentary.pdf]

Supplementary Material

Di Donato, M, Carpintero, Ó. Household food metabolism: Losses, wastes and environmental pressures of food consumption at the regional level in Spain. Foods. Special Issue "Toward Sustainable Food Systems: Approaches and Strategies to Prevent and Reduce Food Loss and Waste".

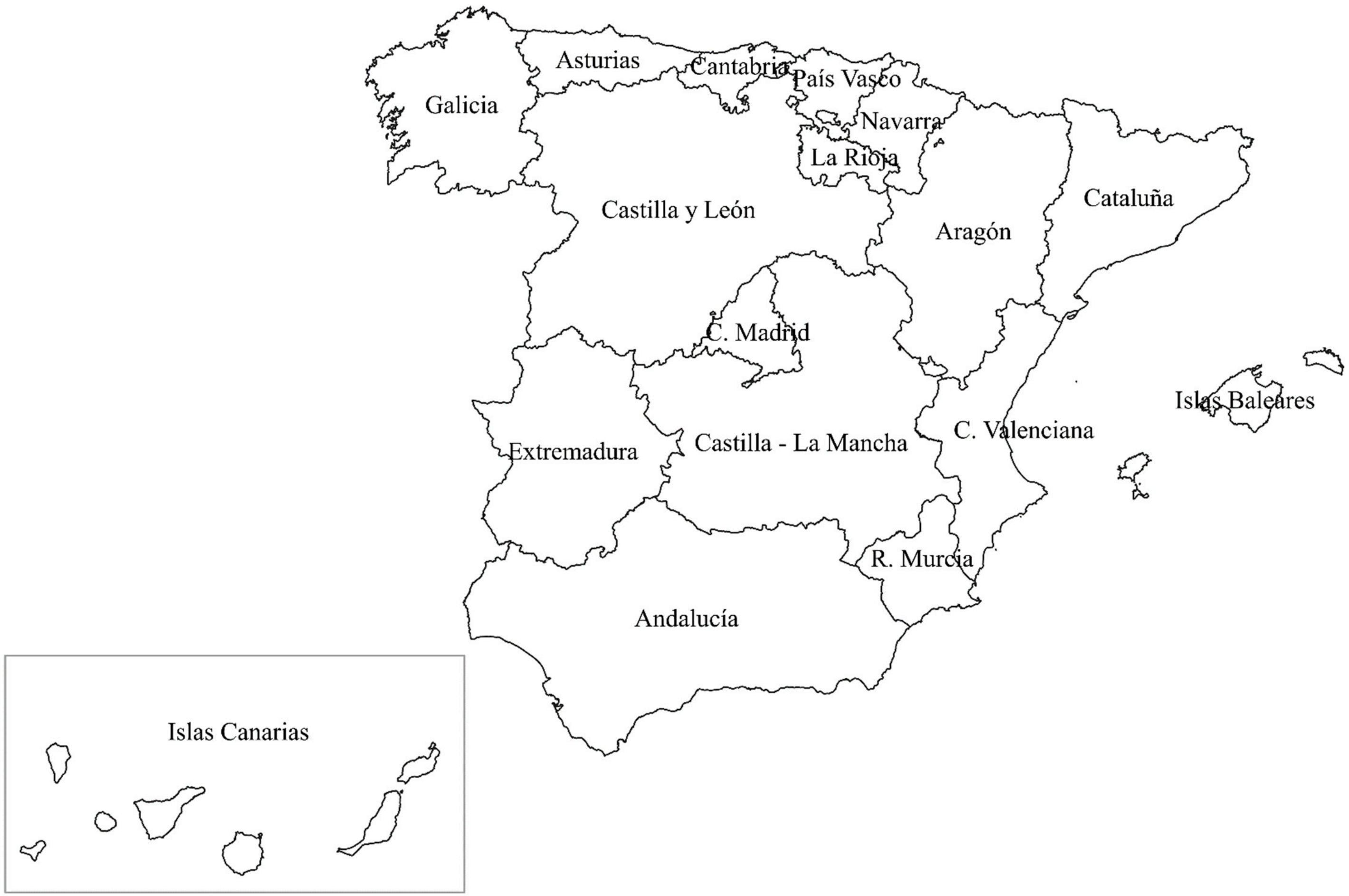

Figure S1. Location and name of EU NUTS-2 regions in Spain.

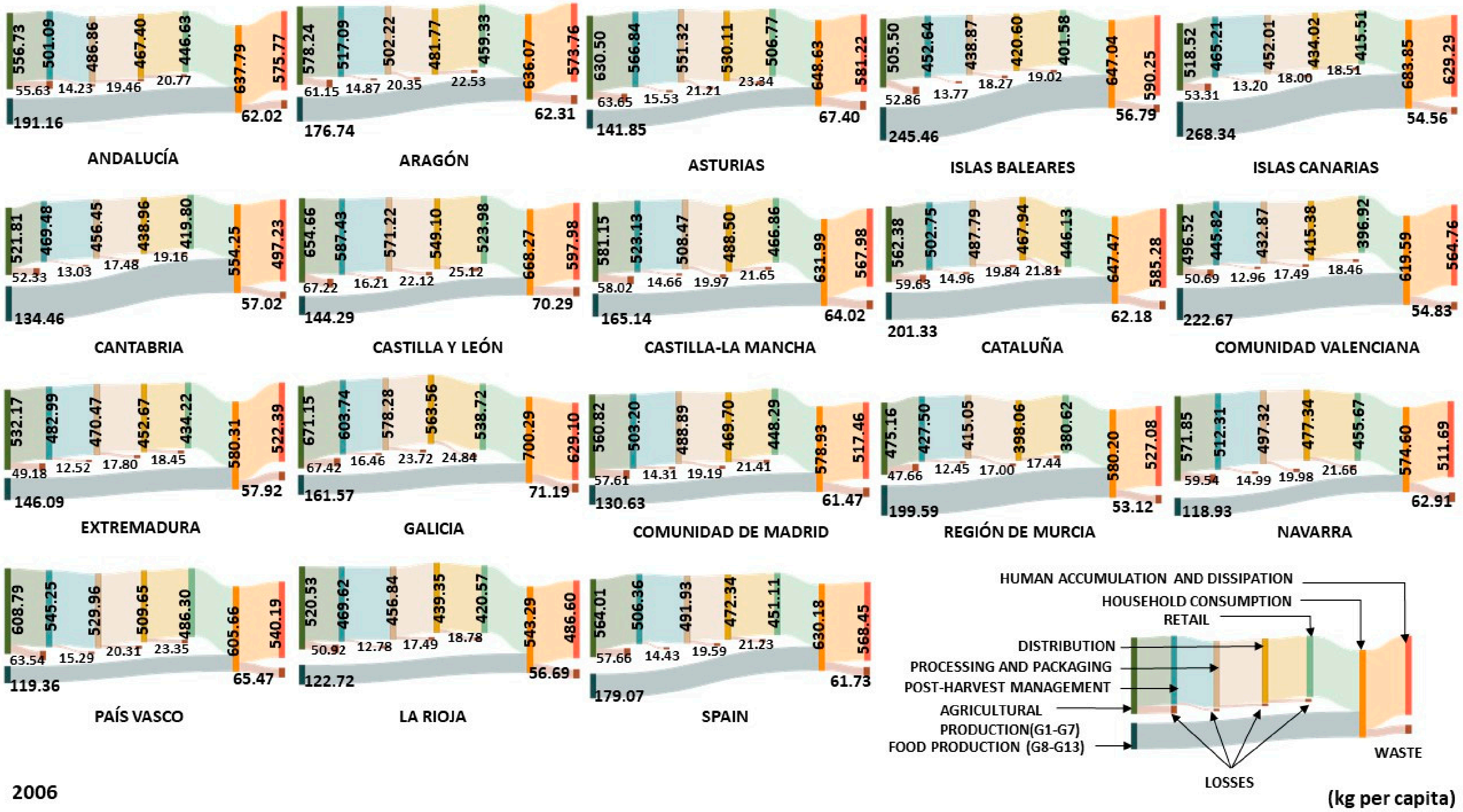

Figure S2. Distribution of losses and waste by food chain phase and NUTS-2 region in Spain (kg per capita) for 2006.

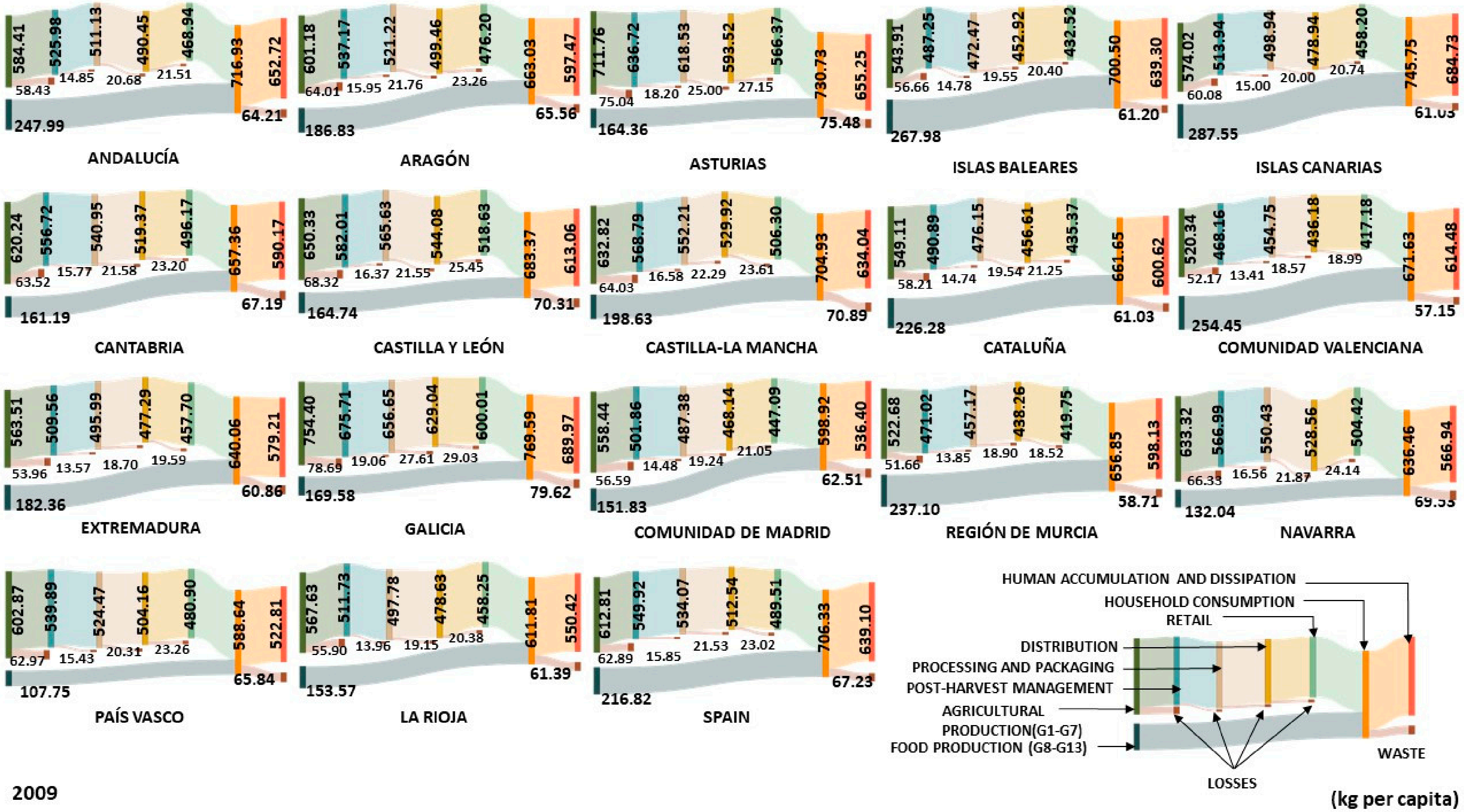

Figure S3. Distribution of losses and waste by food chain phase and NUTS-2 region in Spain (kg per capita) for 2009.

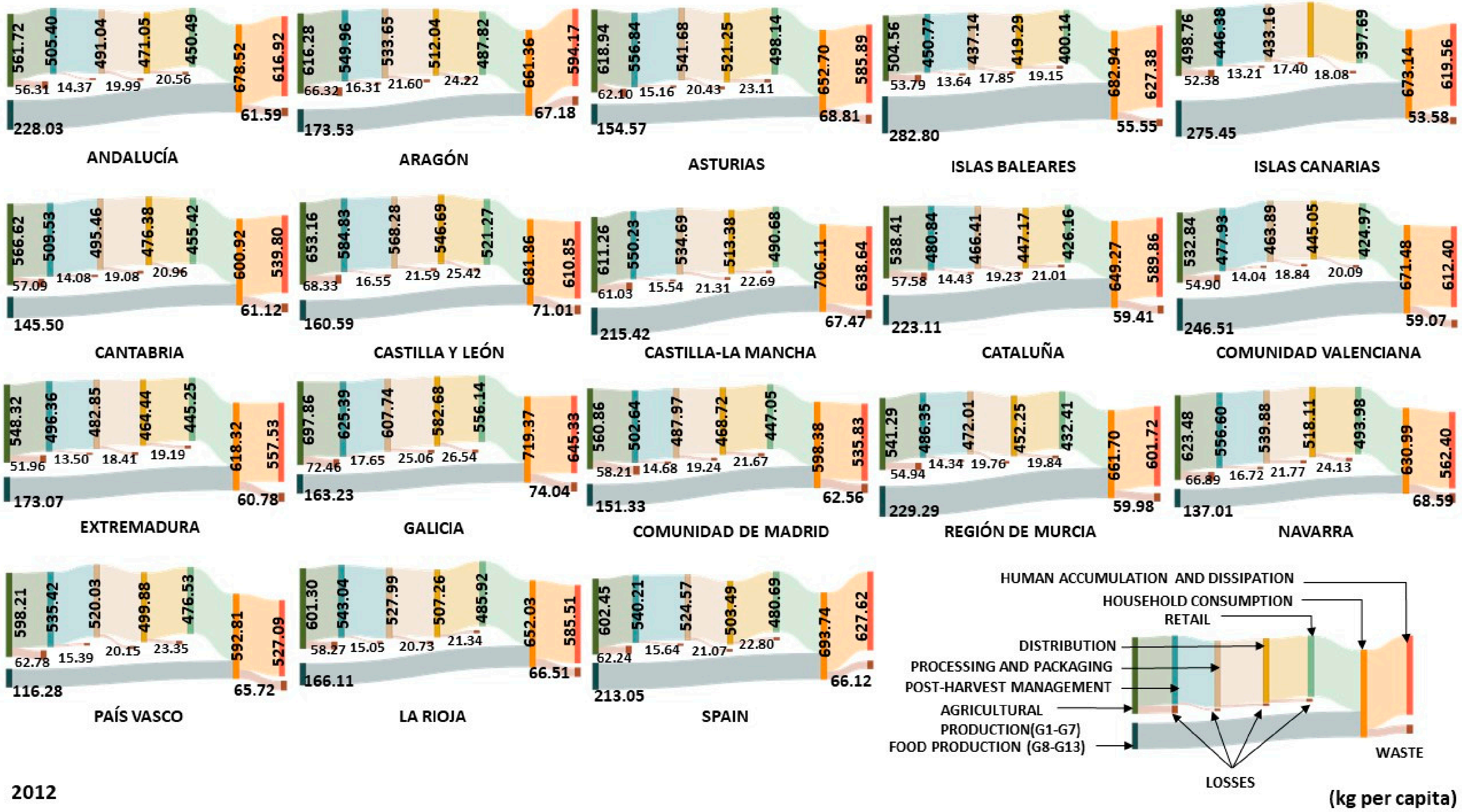

Figure S4. Distribution of losses and waste by food chain phase and NUTS-2 region in Spain (kg per capita) for 2012.

Table S1. Some socio-economic characteristics of NUTS-2 regions in Spain

NUTS-2 REGION	GDP (Euros per capita)			Primary sector share of GAV (%)			Balance of trade* (million Euros)			Population Density (population per km^2)		
	2006	2009	2012	2006	2009	2012	2006	2009	2012	2006	2009	2012
**Andalucía**	17,483	17,573	16,462	5.3%	5.1%	5.0%	-691.2	-8,708.6	2,256.4	94.8	93.2	89.3
**Aragón**	24,239	24,965	23,840	4.6%	4.8%	5.3%	-1,868.1	-1,941.7	-6,503.1	27.5	27.7	26.4
**Asturias**	20,269	20,941	19,954	1.9%	1.3%	1.4%	-867.9	-2,049.7	-2,533.4	99.9	100.5	99.1
**I.Baleares**	25,010	24,561	23,525	0.6%	0.4%	0.5%	-5,214.7	-5,224.8	-6,034.5	219.6	213.7	195.5
**I.Canarias**	20,323	19,810	18,733	1.6%	1.7%	1.9%	-9,230.0	-7,834.1	-7,050.7	278.3	269.7	253.7
**Cantabria**	21,163	21,795	20,585	2.2%	1.5%	1.6%	128.3	-868.6	-1,967.4	109.7	108.9	105.1
**Castilla y León**	20,721	21,509	20,922	5.7%	4.7%	5.1%	-1,827.2	-834.5	-571.0	26.3	26.5	26.1
**Castilla-La Mancha**	18,132	18,586	17,572	7.8%	6.3%	8.2%	-3,041.4	-4,176.9	-2,345.4	26.0	25.7	24.1
**Cataluña**	26,708	26,874	25,806	1.4%	1.2%	1.2%	9,403.3	-7,558.7	-14,792.1	230.3	228.9	217.7
**C.Valenciana**	20,654	20,352	19,038	2.1%	2.0%	2.3%	-3,952.7	-8,977.9	-9,097.8	213.2	212.2	202.0
**Extremadura**	15,128	16,251	15,485	7.4%	6.4%	6.5%	-3,663.0	-2,222.2	-1,876.7	26.1	26.0	25.5
**Galicia**	19,049	20,333	19,514	4.9%	4.5%	5.3%	7,073.9	3,946.4	3,710.0	92.5	92.5	91.1
**C.Madrid**	30,124	31,220	30,349	0.1%	0.1%	0.1%	-24,565.9	37,998.0	-38,718.7	789.7	781.2	736.2
**R.Murcia**	18,966	19,103	18,141	4.5%	3.7%	4.5%	-4,263.4	-6,273.6	-4,297.5	128.0	126.2	118.9
**Navarra**	27,925	28,402	27,030	3.2%	3.1%	3.8%	3,901.5	897.9	-323.6	60.7	59.7	56.2
**País Vasco**	28,225	29,364	28,697	0.8%	0.6%	0.8%	10,300.3	1,654.0	-1,061.0	298.0	297.2	291.4
**La Rioja**	24,307	24,682	23,857	6.8%	5.3%	5.5%	-707.5	191.4	1,119.8	62.4	62.5	59.5

Source: GDP (Regional Accounts of Spain, INE); Primary sector share of GAV (Regional Accounts of Spain, INE); Balance of trade (DATACOMEX database, Ministry of Industry, Tourism and Trade, and C-Interreg Database); Population Density (for population data – EPF (INE); for area – Statistics yearbook (INE)).

(*) Interregional and international flows are included.

Table S2. Aggregation of COICOP/HBS categories used in this work.

COICOP/HBS (4 digits)	COICOP/HBS (5 digits) Categories aggregated	
**0111 Bread and Cereals**	01111	Rice.
	01112	Bread.
	01113	Other bakery products.
	01114	Pasta food.
**0112 Meat**	01121	Fresh, refrigerated or frozen bovine meat.
	01122	Fresh, refrigerated or frozen pork meat.
	01123	Fresh, refrigerated or frozen ovine and caprine meat.
	01124	Fresh, refrigerated or frozen poultry meat.
	01125	Repair and hire footwear.
	01126	Prepared meat and other products containing meat.
	01127	Other edible meats (fresh and frozen).
	01128	Trimmings, fetlock joints and inferior meat cuts.
**0113 Fish**	01131	Fresh or refrigerated fish.
	01132	Frozen fish.
	01133	Fresh, refrigerated or frozen crustacean and molluscs.
	01134	Dry, smoked and salted fish and seafood.
	01135	Other processed or preserved fish and seafood and food made from fish and seafood.
**0114 Milk, cheese and eggs**	01141	Full cream milk.
	01142	Semi skimmed and fully skimmed milk.
	01143	Canned milk.
	01144	Yoghurts and fermented milk.
	01145	Cheese and curd.
	01146	Other milk-based products.
	01147	Eggs.
**0115 Oils and fats**	01151	Butter.
	01152	Margarine and other vegetable fats.
	01153	Olive oil.
	01154	Other edible oils.
	01155	Other animal fats.
**0116 Fruit**	01161	Citrus fruits (fresh, refrigerated or frozen).
	01162	Bananas (fresh, refrigerated or frozen).
	01163	Apples (fresh, refrigerated or frozen).
	01164	Pears (fresh, refrigerated or frozen).
	01165	Fruits with stones (fresh, refrigerated or frozen).
	01166	Olives.
	01167	Berries (fresh, refrigerated or frozen).
	01168	Other fruit (fresh, refrigerated or frozen).
	01169	Dried fruit.
	01160	Prepared fruit and fruit conserve.
**0117 Vegetables**	01171	Leaf or stalk vegetables (fresh, chilled or frozen).
	01172	Cabbages (fresh, chilled or frozen).
	01173	Vegetables cultivated for their fruit (fresh, chilled or frozen).
	01174	Root crops, non-starchy bulbs and mushrooms (fresh, chilled or frozen).
	01175	Dried pulses and vegetables.
	01176	Frozen vegetables.
	01177	Prepared or tinned pulses and vegetables and other products using pulses or vegetables.
	01178	Potatoes.
	01179	Products derived from potatoes, tapioca and other tubes.

Table S2. Aggregation of COICOP/HBS categories used in this work (continuation).

COICOP/HBS (4 digits)	COICOP/HBS (5 digits) Categories aggregated	
**0118 Sugar, jam, honey, chocolate and confectionery**	01181	Sugar.
	01182	Jam, marmalade and honey.
	01183	Chocolate bar.
	01185	Ice Creams.
	01186	Other sugar-based products.
**0121 Coffee, Tea and Cocoa**	01211	Café.
	01212	Tea and infusions.
	01213	Cocoa.
**0122 Mineral water, soft drinks, fruit and vegetable juices**	01221	Mineral water.
	01222	Carbonated or still soft drinks.
	01223	Fruit juices.
	01224	Vegetable juices.
**0211 Spirits and liquors**	02111	Spirits and liquors.
**0212 Wine**	02121	Grape wines and other fermented fruits.
	02122	Other wines.
**0213 Beer**	02131	Beer.

Table S3. Coefficients used to calculate losses and waste associated to consumption of main food categories.

	Consumption	Distribution	Processing and packaging	Post-harvest management	Agricultural production
**G1**	25.00%	2.00%	5.00%	4.00%	2.00%
**G2**	11.00%	4.00%	5.00%	0.70%	3.10%
**G3**	11.00%	9.00%	6.00%	0.50%	9.40%
**G4**	7.00%	0.50%	1.20%	0.50%	3.50%
**G5**	4.00%	1.00%	5.00%	1.00%	10.00%
**G6**	19.00%	10.00%	2.00%	5.00%	20.00%
**G7**	13.33%	6.00%	7.33%	5.00%	16.67%

Source: Adaptation of FAO (2011) coefficients to Spanish HBS categories.

Note: Annex 4 (page 26). Coefficients for Europe (including Russia). G1 = Bread and cereals; G2 = Meat; G3 = Fish and seafood; G4 = Milk, cheese and eggs; G5= Oils and fats; G6 = Fruit; G7 = Vegetables. For G7, the average of roots and tubers, oilseeds and pulses, and fruits and vegetables has been used. There were no available coefficients for categories 8-13 (the conservative assumption of no losses and waste has been adopted in this case).

Table S4. Water footprint of 5-digit COICOP/HBS food categories used (L/kg).

	AND	AR	AS	IBAL	ICAN	CAN	CyL	CLM	CAT	CVA	EXT	GAL	CAM	RMU	NAV	PVA	LR	References
Rice	2219	1968	2064	2064	2064	2064	2064	2002	1882	1905	2248	2064	2064	2039	1847	2064	1757	[1] 110314
Bread	1226	1700	1143	1655	1514	1119	1456	1776	1635	1874	1976	1214	1734	1277	1479	1230	1414	[1] 110100a
Other bakery products	2395	2461	2126	2390	2396	2133	2352	2521	2391	2493	2626	2187	2507	2352	2357	2189	2304	[1] Average flour, eggs, condensed milk, sugar and butter.
Pasta food	1410	1955	1315	1903	1741	1287	1675	2042	1880	2155	2272	1396	1994	1468	1700	1414	1626	[1]110100b
Pastry-cook products	7118	7159	6289	6956	6998	6307	6885	7315	6947	7222	7565	6457	7267	6977	6902	6456	6762	[1] Average flour, sugar and butter.
Sandwich	967	967	967	967	967	967	967	967	967	967	967	967	967	967	967	967	967	[2] 1235 (French)
Other cereal products	1208	1436	976	1356	1339	984	1276	1517	1368	1499	1685	1046	1511	1181	1278	1057	1208	[1] 110220 y 110100 (average)
Bovine meat (F,R,Fr)	19885	19885	19885	19885	19885	19885	19885	19885	19885	19885	19885	19885	19885	19885	19885	19885	19885	[2] 1349, 1350 (Italy)
Pork meat (F,R,Fr)	7184	7184	7184	7184	7184	7184	7184	7184	7184	7184	7184	7184	7184	7184	7184	7184	7184	[3] 020319 (Spain)
Ovine and caprine meat (F,R,C)	9217	9217	9217	9217	9217	9217	9217	9217	9217	9217	9217	9217	9217	9217	9217	9217	9217	[3] 020422, 020423 y 020450 (average) (Spain)
Poultry meat (F,R,C)	4435	4435	4435	4435	4435	4435	4435	4435	4435	4435	4435	4435	4435	4435	4435	4435	4435	[3] 160239 (Spain)
Dr, Sa, Sm meat and offal	6955	6955	6955	6955	6955	6955	6955	6955	6955	6955	6955	6955	6955	6955	6955	6955	6955	[3] 021011 (Spain)
Other pres., proc. and prep meat	15600	15600	15600	15600	15600	15600	15600	15600	15600	15600	15600	15600	15600	15600	15600	15600	15600	[2] 1456 (Spain)
Other edible meat (F,C)	4435	4435	4435	4435	4435	4435	4435	4435	4435	4435	4435	4435	4435	4435	4435	4435	4435	[3] 160239 (Spain)
Trimmings, fetlock joints and inferior meat cuts	15600	15600	15600	15600	15600	15600	15600	15600	15600	15600	15600	15600	15600	15600	15600	15600	15600	[2] 1456 (Spain)

Fish F,R	904	904	904	904	904	904	904	904	904	904	904	904	904	904	904	904	904	[4] Holland
Fr Fish	904	904	904	904	904	904	904	904	904	904	904	904	904	904	904	904	904	[4] Holland
Crustacean and molluscs F,R,Fr	904	904	904	904	904	904	904	904	904	904	904	904	904	904	904	904	904	[4] Holland
Dr,Sa,Sm Fish and seafood	904	904	904	904	904	904	904	904	904	904	904	904	904	904	904	904	904	[4] Holland
Other proc., pres. or prep. fish and seafood	904	904	904	904	904	904	904	904	904	904	904	904	904	904	904	904	904	[4] Holland
Full cream milk	3324	3324	3324	3324	3324	3324	3324	3324	3324	3324	3324	3324	3324	3324	3324	3324	3324	[3] 040130
Semi and fully skimmed milk	1787	1787	1787	1787	1787	1787	1787	1787	1787	1787	1787	1787	1787	1787	1787	1787	1787	[3] 040110
Canned milk	8309	8309	8309	8309	8309	8309	8309	8309	8309	8309	8309	8309	8309	8309	8309	8309	8309	[3] 040221 y 040229 (average)
Yoghurt and fermented milk	2077	2077	2077	2077	2077	2077	2077	2077	2077	2077	2077	2077	2077	2077	2077	2077	2077	[3] 040310
Cheese, curd	9725	9725	9725	9725	9725	9725	9725	9725	9725	9725	9725	9725	9725	9725	9725	9725	9725	[3] 040510 (Spain)
Other milk-based prod.	3329	3329	3329	3329	3329	3329	3329	3329	3329	3329	3329	3329	3329	3329	3329	3329	3329	[3] 040299
Eggs	3674	3674	3674	3674	3674	3674	3674	3674	3674	3674	3674	3674	3674	3674	3674	3674	3674	[3] 040700 (Average)
Butter	9725	9725	9725	9725	9725	9725	9725	9725	9725	9725	9725	9725	9725	9725	9725	9725	9725	[1] 040510
Margarine and other veg. fats	1324	1324	1324	1324	1324	1324	1324	1324	1324	1324	1324	1324	1324	1324	1324	1324	1324	[5] Page 161 (Global)
Olive oil	14000	11977	13324	11031	13324	8820	10863	12588	11296	12358	14026	13324	12216	13078	11269	9275	10571	[1] 150910 y 150990 (Average)
Other edible oils	7447	6589	7007	5994	7007	7007	6150	7000	6149	6352	7644	7007	6794	6870	6039	5066	5921	[1] 151211 y 151219 (Average)
Other animal fats	4435	4435	4435	4435	4435	4435	4435	4435	4435	4435	4435	4435	4435	4435	4435	4435	4435	[1] 020900

Citrus fruits F,R,Fr	487	424	313	400	437	316	387	444	406	438	487	329	432	458	396	338	379	[1] 080510
Bananas F,R,Fr	274	274	178	249	270	189	272	277	239	245	290	188	263	254	257	201	227	[1] 080300
Apples F,R,Fr	578	506	363	468	536	369	470	538	477	509	585	379	519	537	469	396	448	[1] 080810
Pears F,R,Fr	590	516	370	478	547	377	479	549	487	520	597	387	530	548	479	404	457	[1] 080820
Stone Fruits F,R,Fr	690	604	432	560	641	441	559	643	572	610	694	457	620	646	563	472	535	[1] 080930
Olives	2895	2477	2755	2281	2755	1824	2246	2603	2336	2555	2900	2755	2526	2704	2330	1918	2186	[1] 070990aa
Berries F,R,Fr	210	187	138	170	209	139	172	194	178	188	211	142	189	198	172	148	167	[1] 081010
Other fruits F,R,Fr	217	181	129	170	190	134	176	203	170	170	213	138	203	181	169	141	161	[1] 080719
Dr. fruit	4904	4196	2945	3842	4420	2965	3781	4414	3999	4317	4889	3106	4281	4555	3856	3218	3694	[1] 080231 y 080232 (media)
Prep. or cons. Fruit	690	604	432	560	641	441	559	643	572	610	694	457	620	646	563	472	535	[1] 080930
Leaf or stalk vegetables F,R	202	171	130	169	179	138	170	191	163	161	195	139	194	169	161	139	154	[1] 070511
Cabbages F,R	293	250	188	245	261	198	247	278	238	236	285	202	282	248	235	202	225	[1] 070410
Veg. cultivated for their fruit F,R	90	83	66	79	83	63	75	82	81	86	89	69	81	88	77	69	75	[1] 070200
Root crops, non-starchy bulbs and mushrooms F, R	321	266	199	284	283	218	273	309	254	250	306	216	328	260	248	215	236	[1] 071220
Dr pulses and vegetables	3481	3011	2044	2669	3075	2031	2662	3117	2859	3098	3482	2139	3041	3302	2695	2226	2592	[1] 071320
Fr vegetables	513	412	316	433	444	340	421	477	393	390	481	337	500	405	385	338	369	[1] 070820
Prep. or tinned pulses and veg., and other prod. using pulses or veg.	3074	2717	1997	2497	2882	2025	2486	2841	2607	2735	3063	2130	2756	2864	2568	2153	2414	[1] 070920

Potatoes	248	203	149	216	214	162	222	239	198	198	237	168	254	202	192	168	185	[1] 070190
Prod. derived from potatoes, tapioca and other tubes.	925	925	925	925	925	925	925	925	925	925	925	925	925	925	925	925	925	[6] Potato crisps (200 g)
Sugar	1509	1316	897	1192	1251	907	1200	1393	1169	1316	1477	998	1350	1391	1215	985	1143	[1] 170111 y 170191 (average)
Jam, marmalade and honey.	2725	2725	2725	2725	2725	2725	2725	2725	2725	2725	2725	2725	2725	2725	2725	2725	2725	[7]
Chocolate bar	17190	17190	17190	17190	17190	17190	17190	17190	17190	17190	17190	17190	17190	17190	17190	17190	17190	[1] 180500 (global average)
Confectionery prod.	1509	1316	897	1192	1251	907	1200	1393	1169	1316	1477	998	1350	1391	1215	985	1143	[1] 170111 y 170191 (average)
Ice Creams	1903	1903	1903	1903	1903	1903	1903	1903	1903	1903	1903	1903	1903	1903	1903	1903	1903	[7]
Other sugar-based prod.	1509	1316	897	1192	1251	907	1200	1393	1169	1316	1477	998	1350	1391	1215	985	1143	[1] 170111 y 170191 (average)
Coffee	17411	17411	17411	17411	17411	17411	17411	17411	17411	17411	17411	17411	17411	17411	17411	17411	17411	[1] 090111, 090112, 090121 y 090122 (media)
Tea and infusions	8856	8856	8856	8856	8856	8856	8856	8856	8856	8856	8856	8856	8856	8856	8856	8856	8856	[1] 0902
Cocoa	15636	15636	15636	15636	15636	15636	15636	15636	15636	15636	15636	15636	15636	15636	15636	15636	15636	[1] 180310 y 180500 (media)
Mineral wáter	1.39	1.39	1.39	1.39	1.39	1.39	1.39	1.39	1.39	1.39	1.39	1.39	1.39	1.39	1.39	1.39	1.39	[8]
Carbonated or still soft drinks	478	478	478	478	478	478	478	478	478	478	478	478	478	478	478	478	478	[9] Carbonated drinks (169-309 L/0.5 L)
Fruit juices	954	835	616	779	835	625	770	878	794	850	961	642	854	893	779	666	748	[1] 080510, 080540, 080590, 200930, 080810, 080610, 080430 (average)

Vegetable juices	280	259	206	246	259	197	233	257	253	268	277	216	254	275	241	217	235	[1] 070200 (Tomato juice)
Spirits and liquors	1957	1725	1281	1587	1561	1318	1622	1822	1628	1720	1987	1317	1781	1818	1615	1388	1567	[1] 220410
Grape wines and other fermented fruits	1714	1511	1122	1390	1368	1154	1421	1596	1426	1506	1741	1153	1560	1592	1415	1216	1373	[1] 080610
Other wines	1957	1725	1281	1587	1561	1318	1622	1822	1628	1720	1987	1317	1781	1818	1615	1388	1567	[1]080610, sparkling
Beer	324	272	277	257	277	193	261	304	251	268	338	198	296	292	244	204	238	[1]110710

Notes: F = Fresh; R = Refrigerated; Fr = Frozen; Dr=Dried; Sa=Salted; Sm= Smoked; AND= Andalucía; AR=Aragón; AST= Asturias; IBAL=Islas Baleares; ICAN= Islas Canarias; CAN=Cantabria; CyL=Castilla y León; CLM = Castilla-La Mancha; CAT= Cataluña; CVA= Comunidad Valenciana; EXT = Extremadura; GAL= Galicia; CAM=Comunidad de Madrid; RM=Región de Murcia; NAV=Navarra; PVA=País Vasco; LR =La Rioja

References for Table S4

1 Mekonnen, MM; Hoekstra, AY The green, blue and grey water footprint of crops and derived crop products *Hydrol Earth Syst Sci* **2011**, *15*, 1577–1600, doi:105194/hess-15-1577-2011

2 BCFN *Double Pyramid 2016 - A more sustainable future depend on us Technical Database*; BCFN: Parma, Italy, 2016;

3 Mekonnen, M; Hoekstra, A A Global Assessment of the Water Footprint of Farm Animal Products *Ecosystems* **2012**, *15*, 401–415

4 Vanham, D; Mak, TN; Gawlik, BM Urban food consumption and associated water resources: The example of Dutch cities *Sci Total Environ* **2016**, *565*, 232–239, doi:101016/JSCITOTENV201604172

5 Jefferies, D; Muñoz, I; Hodges, J; King, VJ; Aldaya, M; Ercin, AE; Milà i Canals, L; Hoekstra, AY Water Footprint and Life Cycle Assessment as approaches to assess potential impacts of products on water consumption Key learning points from pilot studies on tea and margarine *J Clean Prod* **2012**, *33*, 155–166, doi:101016/jjclepro201204015

6 Hoekstra, AY; Chapagain, AK Water footprints of nations: Water use by people as a function of their consumption pattern In Proceedings of the Water Resources Management; Springer, 2006; Vol 21, pp 35–48

7 Water Footprint Network Product Gallery Available online: https://waterfootprintorg/en/resources/interactive-tools/product-gallery/ (accessed on Feb 19, 2021)

8 IBWA *Water Use Benchmarking Study: Executive Summary*; Alexandria (VI), USA, 2013;

9 Ercin, AE; Aldaya, MM; Hoekstra, AY Corporate Water Footprint Accounting and Impact Assessment: The Case of the Water Footprint of a Sugar-Containing Carbonated Beverage *Water Resour Manag* **2011**, *25*, 721–741, doi:101007/s11269-010-9723-8.

Table S5. Carbon footprint of 5-digit COICOP/HBS food categories used.

5-digit COICOP	Food/Drink	Carbon footprint (kg CO_2-eq/kg)	Ref.	Notes
01111	Rice	2.75	[1]	Reference 20 (Italy)
01112	Bread	0.93	[1]	Reference 7 (Denmark)
01113	Other bakery products	2.25	[1]	Reference 357 (UK)
01114	Pasta food	0.85	[1]	Reference 1264 (Italy)
01115	Pastry-cook products	2.61	[1]	Reference 160 (Uk)
01116	Sandwich	0.55	[1]	Reference 1235 (France)
01117	Other cereal products	0.47	[1]	Reference 1064 (Sweden)
01121	Bovine meat (F,R,Fr)	22.35	[1]	References 1349, 1350 (Italy)
01122	Pork meat (F,R,Fr)	8.70	[1]	Reference 1433 (Spain)
01123	Ovine and caprine meat (F,R,C)	34.03	[1]	Reference 1187 (France)
01124	Poultry meat (F,R,C)	2.46	[1]	Reference 1324 (Portugal)
01125	Dr, Sa, Sm meat and offal	10.70	[1]	Reference 1432 (Spain)
01126	Other pres., proc. and prep meat	17.80	[1]	Reference 1456 (Spain)
01127	Other edible meat (F,C)	4.70	[2]	France
01128	Trimmings, fetlock joints and inferior meat cuts	17.80	[1]	Reference 1456 (Spain)
01131	Fish F,R	6.88	[3]	Page 1610 (Average for different methods)
01132	Fr Fish	9.57	[1]	Reference 1004 (Spain)
01133	Crustacean and molluscs F,R,Fr	8.00	[1]	Reference 1034, 1035 (average)
01134	Dr,Sa,Sm Fish and seafood	4.11	[4]	USA
01135	Other proc., pres. or prep. fish and seafood	6.06	[5]	USA
01141	Full cream milk	1.50	[1]	Reference 1427 (Italy)
01142	Semi and fully skimmed milk	0.98	[1]	Reference 278 (Spain)
01143	Canned milk	3.20	[4]	USA
01144	Yoghurt and fermented milk	2.02	[4]	USA
01145	Cheese, curd	7.49	[1]	Reference 58 (Sweden)
01146	Other milk-based prod.	8.30	[1]	Reference 1351 (Italy)
01147	Eggs	2.27	[1]	Reference 877 (Holland)
01151	Butter	7.20	[1]	Reference 251 (France)
01152	Margarine and other veg. fats	1.66	[1]	Reference 154 (France)
01153	Olive oil	1.20	[1]	Reference 155 (Spain)
01154	Other edible oils	0.80	[1]	Reference 1084 (Portugal)
01155	Other animal fats	11.92	[4]	USA
01161	Citrus fruits F,R,Fr	1.00	[1]	Reference 1068 (Italy)
01162	Bananas F,R,Fr	1.25	[1]	Reference 1281 (Ecuador)
01163	Apples F,R,Fr	0.20	[1]	Reference 1396 (Italy)
01164	Pears F,R,Fr	0.29	[4]	USA
01165	Stone Fruits F,R,Fr	0.36	[4]	USA
01166	Olives	0.63	[1]	Reference 404 (Italy)
01167	Berries F,R,Fr	0.35	[4]	USA
01168	Other fruits F,R,Fr	1.58	[1]	Reference 634 (Italy)
01169	Dr. fruit	1.17	[4]	USA
01160	Prep. or cons. Fruit	1.08	[4]	USA
01171	Leaf or stalk vegetables F,R	0.45	[1]	Reference 34 (Spain)
01172	Cabbages F,R	0.28	[1]	Reference 785 (Holland)
01173	Veg. cultivated for their fruit F,R	1.43	[1]	Reference 232, 346 (Spain) – Average from orchard and greenhouse cultivation
01174	Root crops, non-starchy bulbs and mushrooms F, R	0.17	[1]	Reference 802 (UK)
01175	Dr pulses and vegetables	0.78	[4]	USA

5-digit COICOP	Food/Drink	Carbon footprint (kg CO_2-eq/kg)	Ref.	Notes
01176	Fr vegetables	1.44	[4]	USA
01177	Prep. or tinned pulses and veg., and other prod. using pulses or veg.	1.08	[4]	USA
01178	Potatoes	0.24	[1]	Reference 1402 (Italy)
01179	Prod. derived from potatoes, tapioca and other tubes.	1.44	[4]	USA
01181	Sugar	0.20	[1]	Reference 16 (Brazil) and 373 (Germany)
01182	Jam, marmalade and honey.	2.50	[1]	Reference 1454 (Argentina)
01183	Chocolate bar	3.60	[1]	Reference 604
01184	Confectionery prod.	0.20	[1]	Reference 16 (Brazil) and 373 (Germany)
01185	Ice Creams	2.14	[6]	Unilever ice-cream
01186	Other sugar-based prod.	0.20	[1]	Reference 16 (Brazil) and 373 (Germany)
01211	Coffee	1.20	[7]	Reference Coffee green bean production - arabica (CO_2 land transformation and fossil)
01212	Tea and infusions	0.84	[7]	Reference Tea production, dried
01213	Cocoa	4.98	[8]	Nicaragua
01221	Mineral wáter	0.32	[9]	Still water
01222	Carbonated or still soft drinks	0.17	[10]	Carbonated soft drink. 1 L (1,03 g/mL)
01223	Fruit juice	0.90	[1]	Reference 596 (Italy)
01224	Vegetable juice	0.57	[11]	Turkey
02111	Spirits and liquors	3.91	[12]	Whiskey (USA). 75 cl bottle (Density = 0,939 g/mL)
02121	Grape wines and other fermented fruits	1.83	[13]	(Finland)
02122	Other wines	2.09	[14]	0,75 cl bottle (Density = 0,99 g/mL)
02131	Beer	0.94	[13]	(Finland)

F = Fresh; R = Refrigerated; Fr = Frozen; Dr=Dried; Sa=Salted; Sm= Smoked

References for Table S5

1. BCFN *Double Pyramid 2016 - A more sustainable future depend on us. Technical Database*; BCFN: Parma, Italy, 2016;
2. Clune, S.; Crossin, E.; Verghese, K. Systematic review of greenhouse gas emissions for different fresh food categories. *J. Clean. Prod.* **2017**, *140*, 766–783.
3. Iribarren, D.; Vázquez-Rowe, I.; Hospido, A.; Moreira, M.T.; Feijoo, G. Estimation of the carbon footprint of the Galician fishing activity (NW Spain). *Sci. Total Environ.* **2010**, *408*, 5284–5294.
4. Heller, M.C.; Keoleian, G.A. Greenhouse Gas Emission Estimates of U.S. Dietary Choices and Food Loss. *J. Ind. Ecol.* **2015**, *19*, 391–401, doi:10.1111/jiec.12174.
5. EWG *Meat eater's guide to climate change and health*; Washington D.C., USA, 2011;
6. Zheng, W. *The carbon footprint of ice cream and its mitigating options for Unilever in China*; Groningen, the Netherlands, 2010;
7. Wernet, G.; Bauer, C.; Steubing, B.; Reinhard, J.; Moreno-Ruiz, E.; Weidema, B. The ecoinvent database version 3 (part I): overview and methodology. *Int. J. Life Cycle Assess.* **2016**, *21*, 1218–1230, doi:10.1007/s11367-016-1087-8.
8. Mena Soto, K. *Compendio de experiencias en la mitigación de Gas de Efecto Invernadero (GEI) para la agricultura y ganadería*; Instituto Interamericano de Cooperación para la Agricultura (IICA): San José, Costa Rica., 2015;
9. Jungbluth, N. *Comparison of the environmental impact of drinking water vs. bottled mineral water*; Uster, Switzerland, 2006;
10. BIER *Research on the Carbon Footprint of Carbonated Soft Drinks*; St. Paul (MN), USA., 2012;
11. Karakaya, A.; Özilgen, M. Energy utilization and carbon dioxide emission in the fresh, paste, whole-peeled, diced, and juiced tomato production processes. *Energy* **2011**, *36*, 5101–5110, doi:10.1016/j.energy.2011.06.007.
12. BIER *Research on the Carbon Footprint of Spirits*; St. Paul (MN), USA., 2012;
13. Mattila, T.; Leskinen, P.; Soimakallio, S.; Sironen, S. Uncertainty in environmentally conscious decision making: beer or wine? *Int. J. Life Cycle Assess.* **2012**, *17*, 696–705, doi:10.1007/s11367-012-0413-z.
14. Club Asturiano de Calidad; CITAGRO; CETIEX *Sectores del Vino y la Sidra. Informe final.*; Llanera (Asturias), España, 2013;

Table S6. Parameters of frequency and weight of daily recommended portions, and the resulting annual intake calculated for an omnivorous healthy diet

Food	Portion (crude and net weight)	Recommended frequency	Average intake (kg/person/year)	Notes
Pasta	60-80 g	4-6 daily portions	12.8	Half a portion = 70 g
Bread	40-60 g		65.7	3 portions = 60 g
Rice	60-80 g		12.8	Half a portion = 70 g
Potatoes	150-200 g		73.0	1 portion = 200 g
Vegetables	150-200 g	≥ 2 daily portions	146	400 g/day (2 portions)
Fruit	120-200 g	≥ 3 daily portions	175.2	480 g/day (3 portions of 160 g/day)
Olive oil	10 mL	3-6 daily portions	13.4	4 daily portions (Density = 0,92 g/mL (Charrondiere et al. 2012))
Milk	200-250 mL	2-4 daily portions	75.2	1 portion/day = 200 mL (Density =1,03 g/mL (Charrondiere et al. 2012))
Yoghourt	200-250 g		73.0	1 portion/day (200 g)
Cured cheese	40-60 g		6.0	0,33 portions = 50 g
Fresh cheese	80-125 g		24.7	0,66 portions = 102 g
Fish	125-150 g	3-4 portions/week	28.6	4 weekly portions = 137 g
Lean meat and poultry	100-125 g	Each one 3-4 portions/week	26.0	4 weekly portions = 125 g
Eggs	53-63 g		6.0	2 weekly portions
Fatty meat and inlay	< 50-60 g	Occasional and moderate	2.6	4 monthly portions
Pulses	60-80 g	2-4 per week	10.9	3 weekly portions
Dried fruit	20-30 g	3-7 per week	6.5	5 weekly portions
Water	200 mL	4-8 daily portions	437.6	Density (15ºC) = 0,999 g/mL (Haynes et al. 2016)
Wine	100 mL	1 daily portion (optional and moderate, only for adults)	5.3	Half of the weekends of the year (Density = 1,01 g/mL (Charrondiere et al. 2012))
Beer	200 mL		10.4	Half of the weekends of the year (Densiry = 1 g/mL (Charrondiere et al. 2012))

Source: Weight, portion and recommended frequency (SENC 2004). Annual intakes and assumptions about portions associated (own elaboration).

Table S7. Parameters of frequency and weight of daily recommended portions, and the resulting annual intake calculated for an ovo-lacto-vegetarian healthy diet

Food/Drink	Portion (crude and net)	Recommended frequency	Average intake (kg/persona/year)	Notes
Pasta	40 g	5-8 portions (if pulses inc.)	7.3	Half a portion daily
Bread	60 g		65.7	3 daily portions
Rice	40 g		7.3	Half a portion daily
Potatoes	200 g		73.0	1 daily portion (200 g)
Vegetables	200 g	≥ 2 daily portions	146	2 portions
Fruit	200 g	≥ 3 daily portions	219	3 daily portions
Olive oil	10 mL	3-6 daily portions	13.4	4 daily portions (Density = 0,92 g/mL (Charrondiere et al. 2012))
Milk	200 mL	2 daily portions	75.2	1 daily portion 200 mL (Density = 1,03 g/mL (Charrondiere et al. 2012))
Yoghourt	125 g		22.8	Half a portion daily
Cheese	40-60 g		6.0	Half a portion daily
Eggs	53-63 g	3 weekly portions	4.5	
Pulses	60 g	2 daily portions	6.2	
Dried fruit	20-30 g	3-7 portions/week	5.2	4 weekly portions
Water	200 mL	4-8 daily portions	437.6	Density (15ºC) = 0,999 g/mL (Haynes et al. 2016)
Wine	100 mL	1 daily portion (optional and moderate, only for adults)	5.3	Half of the weekends of the year (Density = 1,01 g/mL (Charrondiere et al. 2012))
Beer	200 mL		10.4	Half of the weekends of the year (Density = 1 g/mL (Charrondiere et al. 2012))

Source: Weight, portion and recommended frequency (Menal-Puey and Marques-Lopes 2017). Annual intakes and assumptions about portions associated (own elaboration).
